# Conserved Nutrient Sensor O-GlcNAc Transferase Is Integral to *C. elegans* Pathogen-Specific Immunity

**DOI:** 10.1371/journal.pone.0113231

**Published:** 2014-12-04

**Authors:** Michelle R. Bond, Salil K. Ghosh, Peng Wang, John A. Hanover

**Affiliations:** 1 National Institute of Diabetes and Digestive and Kidney Diseases, National Institutes of Health, Bethesda, Maryland, United States of America; 2 Center for Biologics Evaluation and Research, Food and Drug Administration, Silver Spring, Maryland, United States of America; 3 Department of Pathology, Medstar Georgetown University Hospital, Washington, District of Columbia, United States of America; University of Birmingham, United Kingdom

## Abstract

Discriminating pathogenic bacteria from bacteria used as a food source is key to *Caenorhabidits elegans* immunity. Using mutants defective in the enzymes of O-linked N-acetylglucosamine (O-GlcNAc) cycling, we examined the role of this nutrient-sensing pathway in the *C. elegans* innate immune response. Genetic analysis showed that deletion of O-GlcNAc transferase (*ogt-1*) yielded animals hypersensitive to the human pathogen *S. aureus* but not to *P. aeruginosa*. Genetic interaction studies revealed that nutrient-responsive OGT-1 acts through the conserved β-catenin (BAR-1) pathway and in concert with p38 MAPK (PMK-1) to modulate the immune response to *S. aureus*. Moreover, whole genome transcriptional profiling revealed that O-GlcNAc cycling mutants exhibited deregulation of unique stress- and immune-responsive genes. The participation of nutrient sensor OGT-1 in an immunity module evolutionarily conserved from *C. elegans* to humans reveals an unexplored nexus between nutrient availability and a pathogen-specific immune response.

## Introduction

Immunity and metabolism are inextricably linked, evolutionarily conserved systems required for the appropriate distinction between nutrient and pathogen. Organisms from *Caenorhabditis elegans* (*C. elegans*) to mammals utilize their innate immune system as a first-line of defense against bacteria, fungi, and viruses [1] with fitness favoring those effectively mounting responses to pathogens. As a versatile model for monitoring pathogen infections [2], *C. elegans’* delineation between bacteria as a nutrient or pathogen is critical as bacteria in decaying organic matter is an essential component of their diet. In this manuscript, we outline data that identify that the evolutionarily conserved, nutrient responsive enzyme O-GlcNAc transferase (OGT-1) is required for *C. elegans* to mount an appropriate innate immune response against select pathogens.

Nutrient flux is governed, in part, by the hexosamine biosynthetic pathway, which serves to produce the nutrient-sensor UDP-N*-*acetylglucosamine (UDP-GlcNAc). 2–5% of glucose is converted to UDP-GlcNAc, which is, in part, used to posttranslationally modify serine and threonine residues with O*-*linked N*-*acetylglucosamine (O-GlcNAc). This posttranslational modification (PTM) plays a role in a variety of cellular processes ranging from protein folding and enzyme activity to cell signaling (for review, see Zachara *et al*. and references therein) [3]. Inappropriate levels of O-GlcNAcylation have been implicated in many human conditions including diabetes, cancer, and neurodegenerative diseases [4]. The enzymes OGT-1 and O-GlcNAcase (OGA-1) govern the addition and removal of O-GlcNAc, respectively. Data from our laboratory [5] and described herein reveal that in animals lacking OGT-1 or OGA-1 activity, stress- and immune-response genes are deregulated. Moreover, it has been shown that mammalian OGT interacts genetically and physically with evolutionarily conserved proteins known to play key roles in the immune response, including β-catenin [6]–[9] and p38 MAPK [10].

While much has been established about the genes involved in the *C. elegans* innate immune response, evidence underscores that mechanisms for pathogen detection and immune response are not fully defined [11]. We suggest that the complicated immune response network in *C. elegans* utilizes OGT-1 in conjunction with other immune system components to respond to pathogens. This “fine tuning” of the innate immune response may rely on O-GlcNAc’s role as a signaling molecule [12].

## Results

The innate immune response in *C. elegans* is complex and has been linked to a number of signaling pathways including insulin signaling (*daf-2* and *daf-16*) [13], [14], MAPK signaling (*pmk-1,* the *C. elegans* p38 MAPK homolog), and β-catenin (*bar-1/egl-5*) [15]. Indeed, OGT has been shown to genetically and/or physically interact with p38 MAPK and β-catenin in mammalian cells [7], [9], [10]. We previously demonstrated genetic interactions between O-GlcNAc cycling and insulin signaling in *C. elegans* [16] and next we sought to determine if interactions also exist between O-GlcNAc cycling and the MAPK and β-catenin pathways. Throughout our work, we took advantage of three well-characterized mutant nematode strains null for either *ogt-1* (*ogt-1(ok1474)* and *ogt-1(ok430)*) or *oga-1* (*oga-1(ok1207)*). In addition, we utilized a new *oga-1* mutant allele (*oga-1(tm3642)*) which recently became available (Figures S1A, B). Without the ability to add or remove O-GlcNAc, respectively, animals lacking OGT-1 activity show complete absence of O-GlcNAc while animals without OGA-1 activity have increased levels of O-GlcNAc by immunoblot (Figure S1C). Multiple *ogt-1* and *oga-1* alleles were used for all assays strengthening our conclusions beyond the statistical power found in assay repetition.

### O-GlcNAc cycling mutants exhibit minimal phenotypes on non-pathogenic bacteria

**Figure 1:**
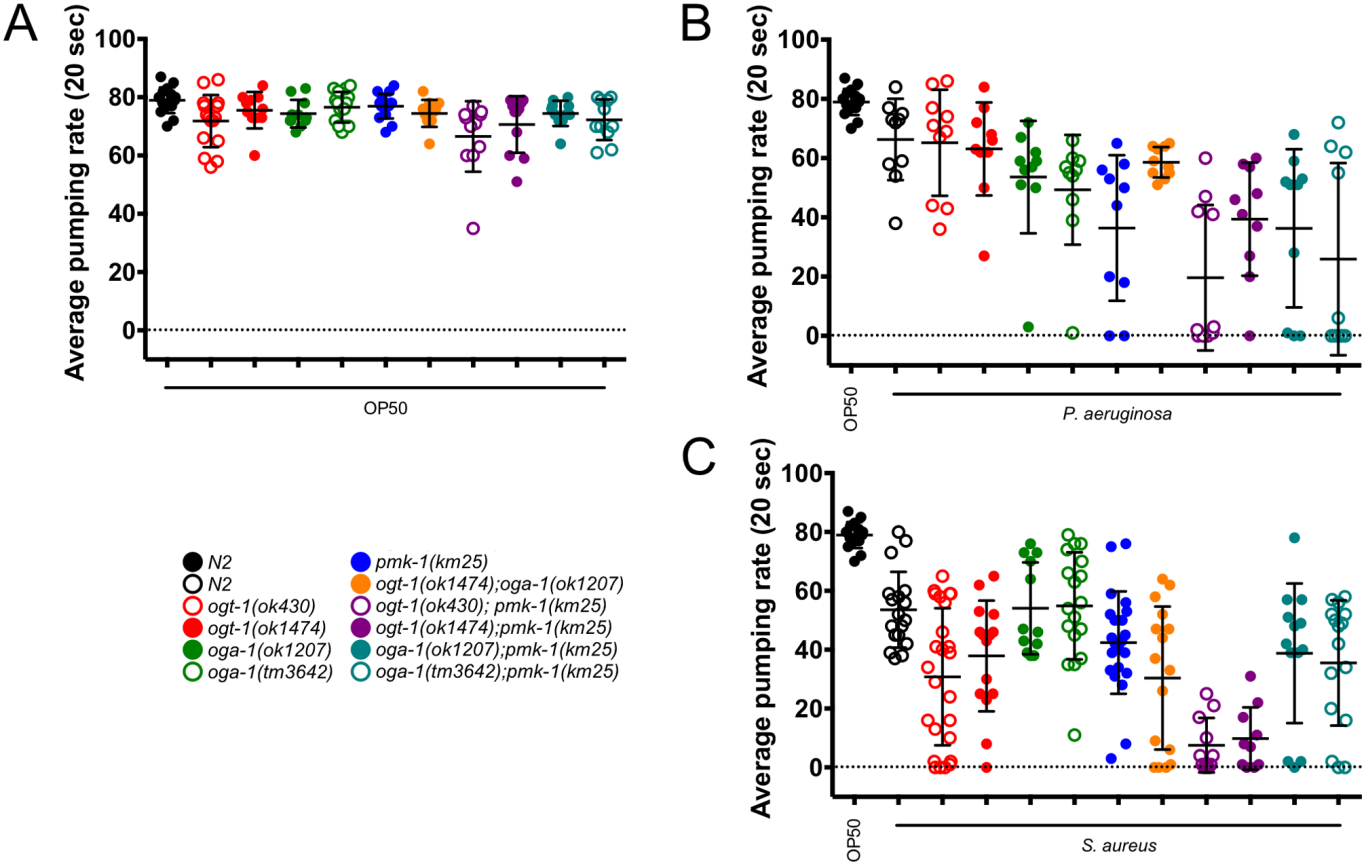
OP50 *E. coli*-fed nematodes are generally healthy while pathogen-fed worms demonstrate a decrease in overall health. (A), (B), and (C) The average pumping rate in a 20 second interval for nematodes fed OP50 or a pathogen. (A) All pumping rates are similar in N2 and mutant animals. (B) The average pumping rate for nematodes fed *P. aeruginosa* is decreased when compared to N2 animals fed OP50. Worms with a *pmk-1(km25)* background exhibit the largest decrease in pumping rate. (C) The average pumping rate for nematodes fed *S. aureus* is decreased when compared to N2 animals fed OP50. Double mutant *ogt-1; pmk-1* animals exhibit the largest decrease in pumping rate. Statistics are available in Table S2 and error bars represent standard deviation. All experiments were performed at 18°C. *n* = 10–25 animals.

Sensitive to bacterial pathogens, *C. elegans* elicit a pathogen-specific immune response as defined by microarray and distinct phenotypes [11]. We began by monitoring *ogt-1*, *oga-1*, and *pmk-1* null animals on OP50, the non-pathogenic laboratory *C. elegans* food source. To summarize multiple individual experiments measuring lifespan, we plotted the median survival data obtained for each of the mutant and double mutant backgrounds. With individual points on the plot representing the results of separate survival curves carried out in triplicate, Figure S2A depicts that all animals exhibited lifespans over 340 hours (∼14 days) after movement to OP50 (Figure S2A, Table S1). Although lifespan values vary slightly from previously reported data, the data in the literature vary as well depending on the lifespan analysis method [5], [17]. For experiments with OP50 and later with *S. aureus*, rather than serial transfer or use of the small molecule FUDR, progeny production was eliminated as a confounding factor with the use of cdc25.1 RNAi. The use of cdc25.1 RNAi has been described by others [15] and prevents the *S. aureus-*induced matricide phenotype. Importantly, all mutants examined exhibited no intestinal distension upon feeding with GFP-labeled OP50 (Figure S3A) and pumping rates similar to wild type (N2) when fed OP50 (pumping rates are considered an indicator of general nematode health; Figure 1, Table S2). Moreover, brood sizes for O-GlcNAc cycling and *pmk-1* null nematodes are within 15% of N2 [16] (Figure S3B and Table S3) suggesting that these non-stressed animals are generally healthy.

### *P. aeruginosa* resistance is not contingent on OGT-1 or OGA-1

Given that OGT interacts with multiple immune modules and perturbed O-GlcNAc cycling alters immune-responsive genes [5], we hypothesized that animals lacking either *ogt-1* or *oga-1* would have decreased survival rates on *P. aeruginosa*. To determine whether O-GlcNAc cycling is required for the *C. elegans* immune response to Gram negative *Pseudomonas aeruginosa* PA14, we monitored survival after pathogen exposure and other phenotypes including pharyngeal pumping. Others have noted that declines in pharyngeal pumping are strongly correlated with age and, more dramatically, with pathogen exposure. Pathogen survival has been shown to increase and pumping rate decline has slowed with treatment of animals with an anti-infective reagent [18], [19]. Pumping rates for animals exposed to *P. aeruginosa* decreased in comparison to N2 animals on OP50 bacteria for all genotypes monitored (Figure 1B, Table S2). In addition, although qualitative, animals fed GFP-labeled PA14 demonstrated varied levels of both accumulation of the fluorescent bacteria and intestinal distension (Figure S3D) [11].

**Figure 2:**
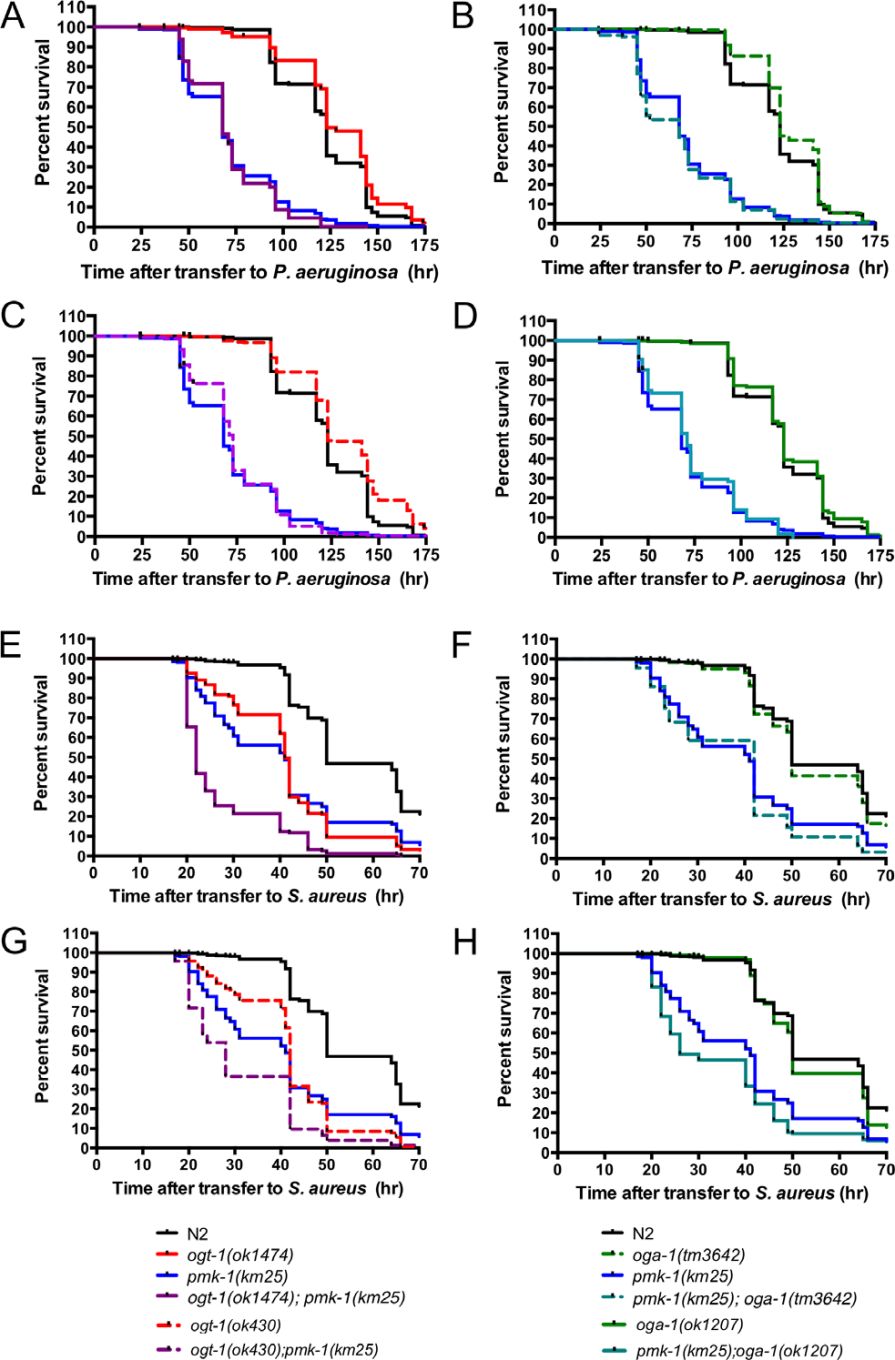
OGT-1 is required for the *C. elegans* response to *S. aureus*. *O-*GlcNAc cycling null nematodes are similarly susceptible to *P. aeruginosa* exposure as N2 animals: (A) *ogt-1(ok1474)*, (B) *oga-1(tm3642)*, (C) *ogt-1(ok430)*, and (D) *oga-1(ok1207)*. (A–H) *pmk-1(km25)* animals are hypersensitive to *P. aeruginosa* and *S. aureus*. (A, C) *ogt-1; pmk-1* and (B, D) *oga-1; pmk-1* are equally susceptible to *P. aeruginosa* as *pmk-1(km25)* single mutants. (E, G) *ogt-1* animals are hypersensitive to *S. aureus* while (F, H) *oga-1* mutants maintain survival similar to N2. (E, G) *ogt-1; pmk-1* are more susceptible to *S. aureus* than either *ogt-1* or *pmk-1* single mutants and (F, H) *pmk-1; oga-1* are similarly susceptible to *S. aureus* as *pmk-1* single mutants. Results are representative of at least two independent assays and are represented by comprehensive plots. *n*≧162. See Table S1 for individual assay statistical analysis.

In line with literature data, we noted that animals lacking PMK-1 activity exhibited a 55% decrease in survival on *P. aeruginosa* (Figure 2A–D). We were surprised to find that mutants null for *ogt-1* and *oga-1* behaved like N2 animals exhibiting median survival indistinguishable from N2 on the Gram negative pathogen (Figures 2A–D, Table S1). To examine the potential genetic interaction of *pmk-1* with *ogt-1* and *oga-1* mutants in *P. aeruginosa* sensitivity, we monitored survival of O-GlcNAc cycling mutants in *pmk-1* null backgrounds. In these genetic epistasis experiments, we would expect that if *ogt-1* and *oga-1* do not modulate the immune response to *P. aeruginosa* double mutants would have median survival values similar to the *pmk-1* single mutants. Indeed, we found that the median survival values for double mutant *ogt-1*; *pmk-1* and *oga-1; pmk-1* animals exposed to *P. aeruginosa* had longevities that were indistinguishable from *pmk-1* single mutants (Figure 2A–D, Table S1) suggesting neither OGT-1 nor OGA-1 are involved in the *C. elegans* innate immune response to *P. aeruginosa*.

### *S. aureus* resistance is modulated by OGT-1

As different innate immunity modulators are thought to govern the unique *C. elegans* response to each pathogen, we hypothesized that O-GlcNAc cycling may play a role in the *C. elegans* susceptibility to Gram positive *Staphylococcus aureus* NCTC8325. Indeed, all strains fed GFP-labeled *S. aureus* exhibited visible bacterial accumulation and intestinal lumen distension by confocal microscopy (Figure S3E) and after 18 hours of exposure to *S. aureus*. In addition, compared to N2 on OP50, all *S. aureus-*exposed genotypes had decreased pharyngeal pumping. Specifically, when compared to N2 on *S. aureus*, all but *oga-1* mutants had statistically decreased pumping rates (Figure 1C, Table S2). These data suggested that *oga-1* mutants were responding to the pathogen in a similar fashion as N2 animals. That the *ogt-1* single and the *ogt-1(ok1474)*; *oga-1(ok1207)* double mutants’ pumping rates both decreased by over 29% when compared to N2 on *S. aureus* suggests an increased sensitivity to *S. aureus* for animals lacking active OGT-1.

Unlike N2 animals, which maintained a median survival of 50 hours after *S. aureus* exposure, *ogt-1(ok430)* and *ogt-1(ok1474)* animals have a 16 and 18% decrease in their survival, respectively (Figures 2E, G, Table S1). These data suggest that the absence of OGT-1 enhanced the nematodes’ susceptibility to killing by *S. aureus*. In contrast, *oga-1* mutant animals appear to exhibit susceptibility comparable to N2 animals (Figures 2F, H, Table S1). Importantly, *ogt-1(ok1474); oga-1(ok1207)* double mutant animals behave like *ogt-1(ok1474)* single mutant animals rather than *oga-1(ok1207)* animals upon *S. aureus* exposure supporting a previously described genetic epitasis (Table S1) [20].

### OGT-1 acts in parallel to PMK-1/p38 MAPK pathway in response to *S. aureus*

With the pathogen sensitivity of the PMK-1 cassette well documented [21]–[23], we aimed to define whether the O-GlcNAc cycling and PMK-1/p38 MAPK pathways intersect in the *C. elegans* innate immune response to *S. aureus*. We found that animals lacking *pmk-1* are exquisitely sensitive to both *P. aeruginosa* and *S. aureus* (Figure 2) also exhibiting decreased pumping compared to N2 animals (Figure 1).

Next, to investigate the interplay between O-GlcNAc cycling and the PMK-1/p38 MAPK signaling cassette, we carried out epistasis analysis. We expected that if *ogt-1* and *pmk-1* were working in different pathways to modulate the immune response to *S. aureus*, we would see an additive effect on the survival curves. While survival of *S. aureus*-fed *pmk-1(km25); oga-1(tm3642)* double mutants were indistinguishable from *pmk-1* single mutants (Figure 2F, Table S1), *ogt-1; pmk-1* nematodes were 32–46% more susceptible to *S. aureus* than either mutant alone (Figures 2E, G, Table S1). This is supported by a striking decrease in pumping rates for the *ogt-1; pmk-1* animals in comparison to N2, *ogt-1*, and *pmk-1* single mutants on *S. aureus* (Figure 1C, Table S2). Our data show a negative correlation between animal survival and pumping rate suggesting that the animals do not simply decrease pathogen intake to survive rather the animals that are succumbing to pathogen exposure most quickly are those that show a decline in pumping rate at an early time point. Interestingly, loss of neither *ogt-1* nor *oga-1* yielded a change in *pmk-1* activation via phosphorylation (Figure S4). The additive sensitivity of *ogt-1; pmk-1* mutants support the notion that OGT-1 acts in parallel to PMK-1 to confer resistance to *S. aureus*.

### Genome-wide transcriptional analysis supports a role for O-GlcNAc in immunity

**Figure 3:**
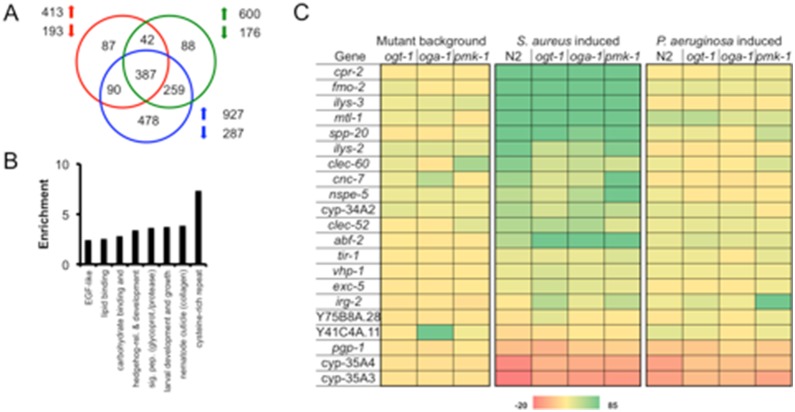
Distinct genes are deregulated in *C. elegans* mutants lacking active OGT-1, OGA-1, or PMK-1 after 6 hours of pathogen exposure. (A) Venn diagram of genes deregulated in *ogt-1(ok1474)* (red), *oga-1(ok1207)* (green), and *pmk-1(km25)* (blue) on OP50 vs. N2. (B) Categories of 387 commonly deregulated genes enriched according to DAVID analysis of microarray samples from A. (C) Heat map of relative levels of genes differentially regulated in mutant backgrounds compared to N2 on OP50 and those deregulated within individual mutants upon pathogen exposure. *cpr-2, fmo-2, ilys-3, clec-60,* and *clec-52* [11], *mtl-1, nspe-5, vhp-1, abf-2,* and *cyp-35A4* [55], *spp-20* and *cyp-35A3* [30], *ilys-2* [11], [28], *cnc-7* [55], [56], *cyp-34A2* [57], *tir-1* [58], [59], *exc-5* [11], [55], *irg-2* [29], Y75B8A.28 [60], [61], Y41C4A.11 [62], *pgp-1* [53].

Previously, we showed that O-GlcNAc cycling is linked to the transcriptional regulation of genes involved in longevity, stress, and immunity [5]. To address the extent to which O-GlcNAc cycling altered the regulation of immune-related genes, we used Affymetrix whole-genome *C. elegans* microarrays to elucidate the changes in *ogt-1* and *oga-1* mutant strains compared to a *pmk-1* mutant strain and wild type animals. The complete list of deregulated genes can be found in Dataset S1. Analyzing L4 larvae on OP50, we noted the more than 2 fold deregulation of 606 (o*gt-1*), 776 (o*ga-1*), and 1214 (*pmk-1*) genes with *p*<0.05 (Figure 3A). While there is overlap between the genes deregulated in these three strains, many deregulated genes are either unique or commonly deregulated with only one other strain supporting the varied responsibilities for each of the missing enzymes. Indeed, when analyzed bioinformatically using DAVID [24], the genes deregulated in all three mutants were predicted to be genes important for immunity (Figure 3B, Table S4). Carbohydrate binding proteins, signal peptides (including glycoproteins), and the nematode cuticle are thought to be involved in the immune response either responding to immune challenge (carbohydrate binding proteins) or protecting the organism from pathogens (the cuticle) [1], [11], [25]–[28]. The deregulation of immunity-associated transcriptional targets shared by *pmk-1, ogt-1,* and *oga-1* prompted us to examine the transcriptional response of the *pmk-1* and O-GlcNAc cycling null strains to the pathogenic bacteria *P. aeruginosa* PA14 (Gram negative) and *S. aureus* NCTC8325 (Gram positive).

We first examined a number of genes considered to be signature pathogen response genes (Figure 3C and references therein) and noted that the different *C. elegans* strains varied in transcriptional response. Using RT-qPCR, we confirmed the microarray data and examined transcript levels of signature *S. aureus*-induced genes including *fmo-2*, a flavin-containing monoxygenase [11] (Figure S5). Indeed, this biomarker was greatly induced after 6 hours of pathogen exposure, albeit at slightly different levels, in only animals exposed to *S. aureus* but not those exposed to *P. aeruginosa* (Figures 3 and S5). The c-type lectin *clec-60* [11] has been suggested to function in an antimicrobial fashion or as a receptor in the *C. elegans* host response and we noted it to be differentially regulated in N2, the single mutants, and the *ogt-1; pmk-1* double mutant. Interestingly, *irg-2*, a p38 MAPK-independent infection response gene [29] was induced above background levels in *ogt-1, pmk-1*, and *ogt-1; pmk-1* animals fed *S. aureus* while N2 and *oga-1* maintained background levels of expression. Overall, pathogen deregulated genes appear distinct, although there is some overlap between genes deregulated by both Gram negative and Gram positive bacteria supporting that the simple bacterivore *C. elegans* mounts a specific, complex innate immune response [30]. The transcriptional profile provides evidence that the response is not starvation dependent and suggests substantial overlap between the *pmk-1* pathogen-response pathway and the signature genes deregulated by loss of function of O-GlcNAc cycling genes.

### *S. aureus* exposed *ogt-1* animals do not exhibit starvation response

Although O-GlcNAc is intimately related to nutrition, our transcriptional data argue that the *S. aureus*-exposed *ogt-1* and *oga-1* worms do not undergo a starvation-induced response. We took advantage of qRT-PCR analysis from the Van Gilst laboratory in which they identified 18 signature starvation-response genes [31]. Of the 18 genes, nine are induced and nine are repressed in starved L4 animals. According to the microarray data collected here, for *ogt-1(ok1474)* animals, only two of nine previously identified fasting-induced genes (*acs-2* and *cpt-3*) were upregulated during *S. aureus* infection and five of nine were repressed (F08A8.4, F08A8.2, *fat-7, acdh-2,* and *lbp-8*) (Figure S6, Dataset S1). Likewise, for *oga-1(ok1207)* animals, only one of nine signature fasting-response genes was upregulated during *S. aureus* infection (*lbp-1*) and three of nine were repressed (*fat-7, acdh-2,* and *lbp-8*). For N2, *ogt-1,* and *oga-1* animals, *hacd-1* was repressed rather than induced upon *S. aureus* infection [11]. In addition, for *ogt-1* animals, *cpt-4* was induced rather than repressed during infection. While we do note that some transcripts were induced or repressed, the magnitudes of the changes observed by transcriptome analysis were modest in comparison to the L4 starved animal dataset. For example, in starved L4 animals, *acs-2* was 39.8 fold induced [31], but in *ogt-1* animals exposed to *S. aureus*, it was induced less than 2.5 fold. Furthermore, of 1873 affytags annotated as upregulated 1.5 fold or greater in starved L1 N2 animals (personal communication, Michael Krause), less than 21% were similarly induced in any strain exposed to *S. aureus* and not necessarily to the same magnitude. Of the 2308 affytags annotated as repressed upon N2 starvation, less than 12% were also downregulated upon *S. aureus* exposure. These data support that *S. aureus*-exposed animals undergo an immunity-based pathogen response rather than starvation-based response.

### Role of OGT-1 and BAR-1 in response to *S. aureus*

**Figure 4:**
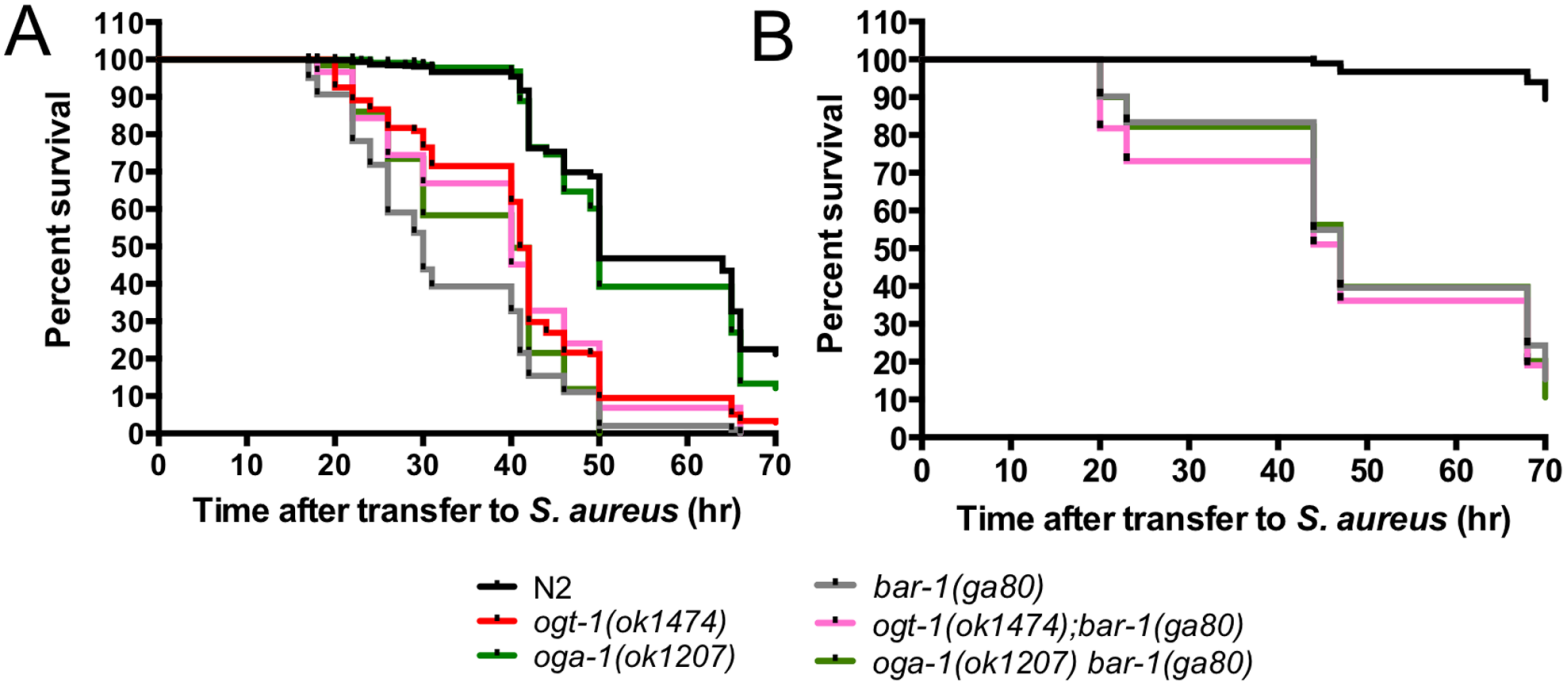
Double mutants ogt-1(ok1474); bar-1(ga80) and oga-1(ok1207) bar-1(ga80) are hypersensitive to S. aureus. (A) *ogt-1(ok1474)* and *bar-1(ga80)* are sensitive to *S. aureus.* (A–B) *ogt-1(ok1474); bar-1(ga80)* and *oga-1(ok1207) bar-1(ga80)* are more sensitive to *S. aureus* than N2 and *oga-1*. Results are representative of at least two independent assays represented by comprehensive plots. *n*≧57. See Table S1 for individual assay statistical analysis and Figure S7 for *ogt-1(ok430); bar-1(ga80)* and *oga-1(tm3642) bar-1(ga80)* data. Animals were exposed to cdc25.1 RNAi for 24 hr (A) or 48 hr (B).

To better define the way in which OGT-1 acts as an immunity modulator, we again used epistasis to determine whether OGT-1 acts in the well-established BAR-1/EGL-5 immunity pathway [15]. Figure 4A shows the increased sensitivity of *ogt-1* and *bar-1* null mutants on *S. aureus* compared to N2 and *oga-1* mutants. In multiple experiments we found that these mutants showed a similar sensitivity to *S. aureus* (Table S1). Since our previous findings suggested that *pmk-1* and *ogt-1* function in distinct pathways, we next focused on the interactions of *bar-1* with *ogt-1* and *oga-1.* Investigation of the interplay between the BAR-1 immunity pathway and OGT-1 revealed that loss of either *ogt-1* or *oga-1* in a *bar-1* background yielded animals almost equally sensitive to *S. aureus* when compared to either the *bar-1* or *ogt-1* single mutants suggesting that these enzymes function in the same pathway to promote *S. aureus* pathogen resistance (Figure 4A, Table S1). Because of the potential importance of this finding, we sought to further verify the survival curves for these double mutants using slightly different experimental conditions with longer pre-pathogen treatment with cdc25.1 RNAi. In these experiments, N2 animals showed less sensitivity to *S. aureus* exposure thus making the assay more sensitive to small changes in mutant survival. These studies also employed two independent alleles of *ogt-1* or *oga-1* with only minor allele-specific differences emerging in the double mutants (Figures 4B, S6, Table S1). The median survival of the double mutants under these conditions was almost identical to the *bar-1* mutants alone under these conditions. The results obtained under these distinct conditions provide strong support the conclusion that *ogt-1* and *bar-1* function in the same pathway (Figures 4B, S6).

As a control for these studies, we examined the genetic interactions between *bar-1* mutants and O-GlcNAc cycling mutants in modulating normal lifespan. Unlike *ogt-1* animals that appear to have normal lifespans on OP50, *bar-1* animals have decreased lifespans (Figures S2A, B, Table S1) both without and with elimination of progeny production [15], [32], morphological phenotypes [33], [34], and decreased fecundity (Figure S3C, Table S3). Double mutant *ogt-1; bar-1* and the *oga-1(tm3642) bar-1(ga80)* double mutant animals fed OP50 have lifespans that decrease further than the *bar-1*, *ogt-1*, and *oga-1* single mutants (Figure S2B and Table S1). In contrast, the *oga-1(ok1207) bar-1(ga80)* double mutant has a lifespan similar to the *bar-1(ga80)* single mutant. Thus, the O-GlcNAc cycling mutants show complex genetic interactions with *bar-1* mutants in regulating lifespan on OP50. These genetic interactions are likely distinct from those observed above for sensitivity to *S. aureus*.

**Figure 5:**
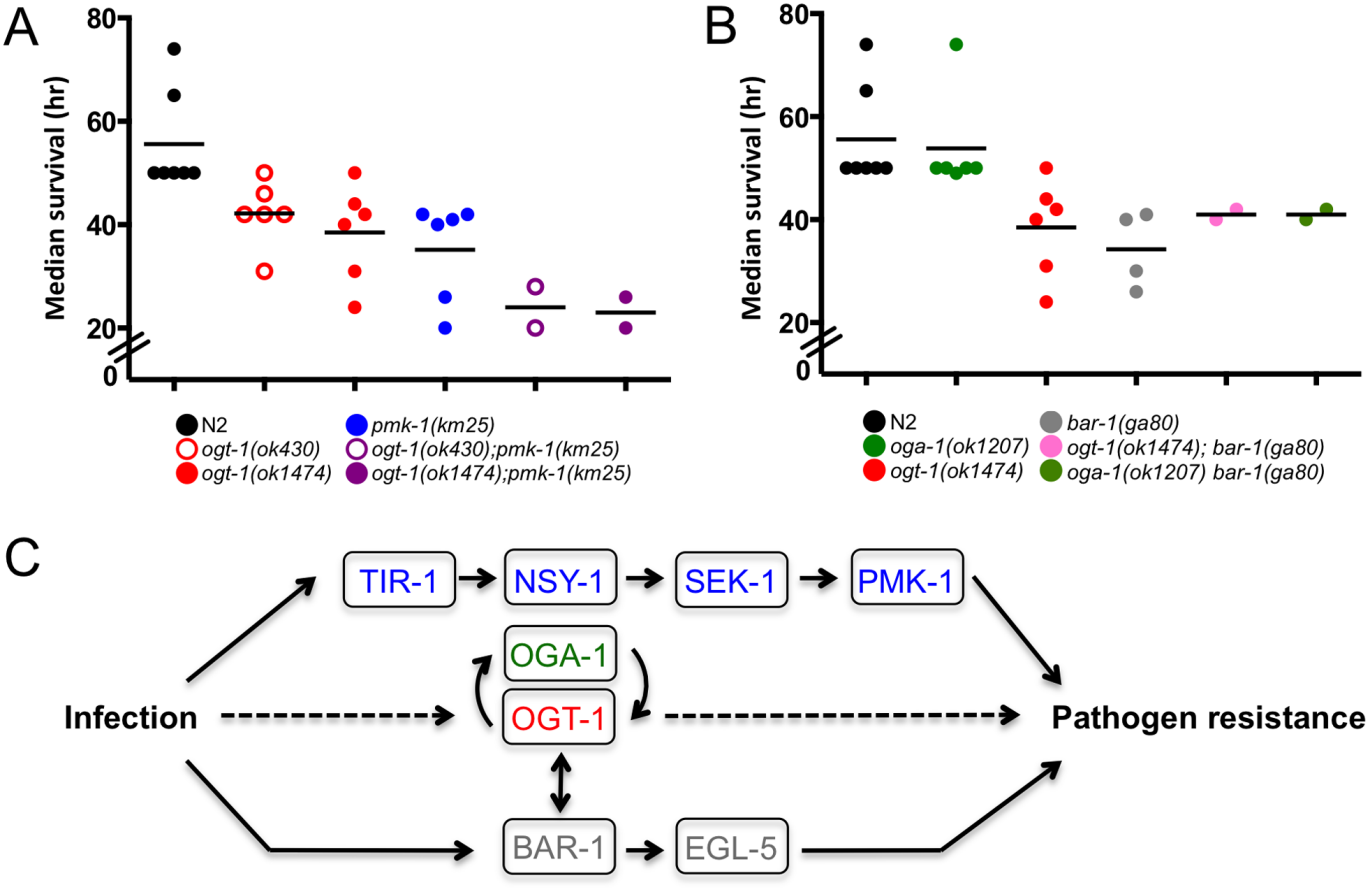
*S. aureus* exposed *C. elegans* exhibit differential sensitivity to *S. aureus*. (A–B) Median survival values (hours after transfer to *S. aureus*) representing at least two independent biological replicates. Each circle represents the median survival for an individual assay done in technical duplicate or triplicate, statistics are available in Table S1. *ogt-1; pmk-1* are more sensitive than N2 and single mutants to *S. aureus* while the *ogt-1; bar-1* mutants are similarly susceptible as the *bar-1* single mutant. (C) Simplified model showing how OGT-1 participates in the *C. elegans* immune response.

To summarize the data from multiple individual experiments performed under the same conditions measuring survival upon exposure to *S. aureus*, we plotted the median survival data for each of the mutant backgrounds (Figures 5A, B). Note that each of the individual points on this plot represents the results of separate survival curves carried out in at least duplicate. Exposure to pathogenic *S. aureus* yields *ogt-1; pmk-1* double mutants that have a decrease in survival when compared to their single mutant counterparts (Figure 5A). Under the same conditions *ogt-1; bar-1* double mutants have median survival values similar to mutants lacking *ogt-1* or *bar-1* (Figure 5B). Together, these data support a model in which OGT-1 acts through the established BAR-1/EGL-5 immunity pathway in parallel with the PMK-1/p38 MAPK pathway to modulate the *C. elegans* immune response to *S. aureus* (Figure 5C).

## Discussion

Growing clinical problems ranging from obesity and heart disease to drug resistant bacteria highlight the importance for understanding how nutritional cues and other environmental factors impact the host immune response. Burgeoning data suggest that the PTM O-GlcNAc is directly related to nutritional intake and acts as a signaling molecule in a variety of biological processes [4]. Recent papers outline that appropriate expression of OGT and p38 MAPK are required for the immune response to lipopolysaccharide [35] and cytokine release may be contingent upon appropriate O-GlcNAcylation [36]. In addition, microarray data (from [5] and this manuscript) suggest that loss of O-GlcNAc in *ogt-1* mutants or increased O-GlcNAc in *oga-1* mutants yields changes in stress- and immune-responsive genes. Furthermore, research from multiple groups delineates that the interaction partners of OGT include proteins involved in the immune response [8]–[10]. As both *S. aureus* and *P. aeruginosa* accumulate in the nematode intestine [37] and elicit strong transcriptional responses [11], we elected to monitor the immune response to two distinct pathogens^.^ Direct genetic evidence described herein supports a novel biological role for the evolutionarily conserved, nutrient sensitive OGT-1 in the *C. elegans*’ innate immune response to select bacterial pathogens.

### OGT-1 is involved in a pathogen-specific immune response pathway

We initially hypothesized that loss of either OGT-1 or OGA-1 would yield changes to pathogen response, and we were intrigued to find that while O-GlcNAc cycling mutants are viable and healthy under normal laboratory conditions, only *ogt*-1 null animals had a striking decrease in viability in comparison to N2 animals when fed *S. aureus*. In addition, *ogt-1* animals did not have a decrease in viability on *P. aeruginosa* and *oga*-1 null animals maintain a similar longevity to N2 animals on both pathogenic bacteria. That *ogt-1* animals have an immunodeficiency on *S. aureus* while *oga-1* animals survive pathogen exposure at a rate similar to N2 suggests that it is the presence of O-GlcNAc rather than simply the global quantity that influences the immune response. Indeed, Love *et al.* also describe a sensitivity to UV stress in *ogt-1(ok430)* nematodes that is absent in *oga-1(ok1207)* nematodes [5]. Together, these data support the notion that inappropriate O-GlcNAcylation of specific, key OGT-1 targets leads to this immunodeficiency as the presence or absence of the PTM can dramatically affect protein properties including localization and activity (see Zachara *et al.* and references therein [3]). Alternatively, the non-catalytic functions of OGT-1 involving protein-protein interactions [38] may be important for immune regulation. Analysis of alleles with mutations affecting only O-GlcNAc transferase catalytic activity may be possible in the future to address this outstanding question.

### OGT-1 animals exhibit immune-related transcriptional response

The transcriptional data reported herein strongly argue that *ogt-1* animals have a defective immune response rather than a starvation response upon pathogen exposure. We note that when compared to microarray data from starved animals collected by the Van Gilst [31] and Krause (personal communication) laboratories, the number and magnitude of starvation-response genes for the *S. aureus* exposed animals was negligible (Figure S6). Indeed, evidence discussed by Sifri *et al.* describes that pathogens shorten longevity by actively killing worms [39]. Moreover, it has been established that mutations in microorganisms that attenuate their pathogenesis in mammalian hosts yield decreased *C. elegans* killing ([39] and references therein). Interestingly, although heat-killed *S. aureus* is of reasonable food *quality*, it activates a similar transcriptional response as live *S. aureus* suggesting the host response to *S. aureus* may be mediated by pathogen-associated molecular patterns [11]. Together these data support a model that all strains examined herein exhibit an immunity-based pathogen response.

### OGT-1 exhibits complex genetic interactions with other immunomodulatory pathways

Pathways with known involvement in *C. elegans* innate immunity include the insulin-like signaling pathway, the MAPK pathway, and the β-catenin/Hox gene pathways [13], [14] [15]. We, and others, showed that *ogt-1* and *oga-1* genetically interact with the insulin-like signaling pathway in *C. elegans* impacting longevity and dauer phenotypes [20], [40]. While others have shown that mutants lacking insulin-like signaling through *daf-2* appear to be pathogen resistant [14], [15], we demonstrate that *ogt-1* mutants exhibit an enhanced sensitivity to *S. aureus* infection. Therefore, we focused our genetic efforts on epistasis experiments with *pmk-1* and *bar-1* null animals. Previous work and the survival curves as well as other phenotypes described herein demonstrate that *pmk-1* and *bar-1* animals have a decreased survival on at least *S. aureus* bacteria (Figures 2, 4, and S6) and different pathways regulate the *C. elegans’* response to each pathogen (Figures 3 and S3). Indeed, loss of these key enzymes causes sensitivity to bacteria, fungus, and/or other stressors in *C. elegans* [11], [15], [23]. Moreover, the genes induced in response to *S. aureus* are largely distinct from those induced by *P. aeruginosa* ([11] and array data in this manuscript) indicating that our understanding of the mechanisms for pathogen detection and immune response in *C. elegans* has yet to be fully realized.

### Genetic evidence suggests *ogt-1* and *pmk-1* function in parallel pathways to modulate immunity

Two lines of evidence suggest that *ogt-1* and *pmk-1* function in parallel pathways to modulate innate immunity. First, *pmk-1* mutants exhibit sensitivity to both *P. aeruginosa* and *S. aureus* whereas *ogt-1* mutants show selective sensitivity to *S. aureus*. Second, genetic epistasis experiments reveal that the decrease in survival for *ogt-1*; *pmk-1* mutants is additive when compared to individual *ogt-1* and *pmk-1* null alleles. These findings suggest that OGT-1 and PMK-1 have distinct roles and act in concert to promote a pathogen specific immune response with OGT-1 having an important function in pathogen-specific host defense while PMK-1 is likely important for a general immune response [11], [23], [41]. Importantly, although Irazoqui *et al.* note that BAR-1 is the critical regulator of the *S. aureus* pathogen response, their survival data support that an absence of *pmk-1* also yields increased susceptibility to *S. aureus* [15]. Importantly, our survival, healthspan, and transcriptional data on both pathogen and OP50 support that the enhanced pathogen susceptibility for *ogt-1* and *pmk-1* animals is not due to a non-specific decrease in viability or poor nutrition. The differences between the nematode immune response to the two pathogenic bacteria is a testament to not only the complexity of the *C. elegans* innate immune system but is also indicative of the complex role OGT-1 and OGA-1 play in many biological processes including fertility as described here and elsewhere [16], [20]. These data are intriguing as the human p38 MAPK maps to the catalytic domain of hOGT [10] and high-throughput two hybrid analyses in *C. elegans* suggests a link between these two enzymes and their activity and/or substrates [6].

### OGT-1 and BAR-1 function in same pathway to mediate immunity to *S. aureus*

The β-catenin homolog *bar-1* has been shown to participate in a signaling cascade linked to the activation of the posterior Hox gene *egl-5* that is critical to innate immunity in *C. elegans* [15]. Interestingly, *bar-1* and *ogt-1* have been shown to interact genetically [6]. The *C. elegans egl-5* is a Hox gene homologous to the *Drosophila* gene *Abdominal-B* [42], [43] and is part of the six-gene Hox gene cluster on chromosome III. Intriguingly, *ogt-1* is also tightly linked to this Hox gene cluster sitting next to *lin-39*. Both *ogt-1* and *bar-1* mutants were found to be more susceptible to infection by *S. aureus* than N2 animals. For genetic epistasis experiments, we expected that if two enzymes acted in the same pathway to modulate the immune response, the double mutant would have the same phenotype as the individual mutants. We observed that both *ogt-1; bar-1* and *oga-1 bar-1* double mutants exhibit a decreased survival similar to single mutants upon *S. aureus* exposure (Figures 4 and S6). These data suggest that *ogt-1* may act in the same pathway as *bar-1* to generate a *S. aureus* immune response. Thus, our genetic and transcriptional findings point to an evolutionarily conserved link between *ogt-1* and *bar-1* regulation of immunity.

### OGT-1 is an integrator of immune signaling

In mammals, O-GlcNAc has been implicated in a number of aspects of the host immune system: it is involved in altering the motility of neutrophils [44], in activating T and B lymphocytes [45], and mediating stimulus-specific activation of NF-κB-dependent transcription. It is possible that without OGT-1, proteins lacking O-GlcNAc are inappropriately expressed, localized, or activated leading to a poorly-orchestrated immune response. We suggest that OGT-1 plays a critical role in the *C. elegans* innate immune response to *S. aureus* and may be a key component for immune regulation operating within the *bar-1* signaling pathway (Figure 5C). Our findings provide the first insight into OGT-1’s role in the *C. elegans* immune response and highlight that our knowledge of signaling pathways involved in innate immunity is likely incomplete. We expect future work will identify the OGT-1 specific targets involved in the signaling pathways associated with pathogen response. These data are expected to have implications for our understanding of the conserved mechanisms by which animals respond to intestinal pathogen assault and may yield fruitful information about potential therapeutic targets.

## Materials and Methods

### Bacterial strains

Bacterial strains used in this study include *E. coli* strain OP50, *E. coli* strain OP50-GFP, *E. coli* strain HT115, *S. aureus* strain NCTC8325 [37], [46], *S. aureus* strain *S. aureus*-GFP [47], *P. aeruginosa* strain PA14 [48], and *P. aeruginosa* strain PA14/GFP [48]. All strains were maintained at −80°C in their appropriate medium containing 15% glycerol. The *E. coli* OP50 strain [49] was cultured at room temperature (RT) in Luria-Bertani (LB) broth without antibiotic. Bacteria were plated on nematode growth media (NGM), replacing the Bactopeptone with Bactotryptone. NGM plates contained: 1 L ddH_2_O, 2 g tryptone, 20 g agar, 3 g NaCl, 1 mL 1 M CaCl_2_, 1 mL 5 mg/mL cholesterol, 1 mL 1 M MgSO_4_, 25 mL 1M KPO_4_. M9 buffer was composed of: 1 L ddH_2_O, 3 g KH_2_PO_4_, 6 g Na_2_HPO_4_, 5 g NaCl, 1 ml 1 M MgSO_4_.

HT115 *E. coli* carrying dsRNA were streaked onto LB plates containing ampicillin (100 µg/mL) and tetracycline (25 µg/mL). RNAi clones were obtained from either the Ahringer or Open Biosystems Libraries and identity was confirmed by sequencing. Liquid cultures in LB with 50 µg/mL AMP were grown overnight at 37°C with shaking. Bacteria were plated on NGM plates containing 25 µg/mL AMP and 1 mM IPTG.

The *S. aureus* NCTC8235 strain was cultured overnight at 37°C with aeration in tryptic soy broth containing 10 µg/mL nalidixic acid. The *S. aureus*-GFP strain was similarly cultured with the addition of 10 µg/mL chloramphenicol to maintain the plasmid. 50 µL of *S. aureus* strains were then seeded directly onto tryptic soy agar (TSA) plates containing the appropriate antibiotics and incubated for 3–6 hours at 37°C for pathogen infection assays. Plates were cooled 30–60 min prior to the addition of nematodes.

*P. aeruginosa* strains were cultured in King’s Broth containing 100 µg/mL rifampicin. The PA14/GFP strain was similarly cultured with the addition of 300 µg/mL carbenicillin. 50 µL of *P. aeruginosa* strains were seeded directly onto 0.35% peptone NGM plates containing the appropriate antibiotics and incubated overnight at 37°C for pathogen infection assays. Plates were cooled prior to the addition of nematodes. NGM with 0.35% peptone: 250 mL ddH_2_O, 0.875 g peptone, 5 g agar, 0.75 g NaCl, 0.25 mL 1 M CaCl_2_, 0.25 mL 5 mg/mL cholesterol, 0.25 mL 1 M MgSO_4_, 6.25 mL 1 M KPO_4_.

### C. elegans strains

All *C. elegans* strains were maintained on NGM plates supplemented with *E. coli* OP50 as a food source [49] and maintained at 16°C unless otherwise noted. Nematodes were manipulated using standard techniques [50]. N2, *ogt-1(ok1474)*, *ogt-1(ok430)*, *oga-1(ok1207)*, *oga-1(tm3642)*, *pmk-1(km25)* [22], and *bar-1(ga80)* [51], [52] were used in the experiments described in this paper. The *pmk-1(km25)* and *oga-1(tm3642)* strains were obtained from the *C. elegans* Gene Knockout Consortium and Shohei Mitani, Tokyo Women’s Medical College, Tokyo, Japan, as viable homozygotes, respectively. All strains were backcrossed to N2 laboratory strain a minimum of four times prior to study, with the exception of *oga-1(tm3642)* which was backcrossed a minimum of twice. Upon simple inspection, all single mutants appeared phenotypically normal with the exception of *bar-1(ga80)*X which has several vulval phenotypes [51], [52].

To generate double mutant strains, the presence of the deletion alleles were verified using nested PCR primers as described by the *C. elegans* Gene Knockout Consortium. Primers for *ogt-1(ok430)*, *ogt-1(ok1474)*, *oga-1(ok1207)*, and *pmk-1(km25)* have been described elsewhere and are available upon request. The deletion allele *oga-1(tm3642)* was tracked by nested PCR using the outside pair of primers MWK 555 (5′-gtggatgtctcctgctagctgttctccg-3′) and MWK 552 (5′-gtgacggccacgagactatctccg-3′) and the inside set of primers MWK 556 (5′-ctggggtccatggacgtccgtagaagcctg-3′), MWK 554 (5′-cttttcccggcgagattgcatacac-3′) and MWK 553 (5′-gtggccgtggagacagggactagg-3′). The single nucleotide polymorphism in *bar-1(ga80)* strain yields a presumptive early stop codon and the SNP was monitored by sequencing.

The following double mutant strains were constructed for this study using standard genetic techniques: *ogt-1(ok1474); oga-1(ok1207), ogt-1(ok1474); pmk-1(km25), ogt-1(ok430); pmk-1(km25), pmk-1(km25); oga-1(ok1207), pmk-1(km25); oga-1(tm3642), ogt-1(ok430); bar-1(ga80), oga-1(ok1207) bar-1(ga80), ogt-1(ok1474); bar-1(ga80), oga-1(tm3642) bar-1(ga80)*.

### Gene accession information

Gene Public Name, Gene WormBase ID: *ogt-1,* WBGene00003858; *oga-1,* WBGene00020596; *bar-1*, WBGene00000238; *pmk-1*, WBGene00004055; *sek-1*, WBGene00004758; *egl-5*, WBGene00001174; *cpr-2*, WBGene00000782; *fmo-2*, WBGene00001477; *ilys-3*, WBGene00016670; *mtl-1*, WBGene00003473; *spp-20*, WBGene00005005; ilys-2, WBGene00016669; *clec-60*, WBGene00014046; *cnc-7*, WBGene00010005; *nspe-5*, WBGene00012594; cyp-34A2, WBGene00011699; *clec-52*, WBGene00015052; *abf-2*, WBGene00000013; *tir-1*, WBGene00006575; *vhp-1*, WBGene00006923; *exc-5*, WBGene00001366*; irg-2*, WBGene00016783; Y75B8A.28, WBGene00013559; Y41C4A.11, WBGene00012757; *pgp-1*, WBGene00003995; cyp-35A4, WBGene00016786; cyp-35A3, WBGene00019565; *acs-2*, WBGene00009221; *cpt-3*, WBGene00021703; F08A8.4, WBGene00008567; F08A8.2, WBGene00008565; *fat-7*, WBGene00001399; *acdh-2*, WBGene00015894; *lbp-8*, WBGene00002260; *lbp-1*, WBGene00002253; *hacd-1*, WBGene00019978; *cpt-4*, WBGene00019644.

### Pathogen infection assays

For OP50 and *S. aureus* survival experiments, synchronized L4 nematodes were first placed on RNAi plates seeded with cdc-25.1 dsRNA-expressing *E. coli* HT115 in order to sterilize animals prior to the killing assays [11]. L4 animals were fed RNAi for 24 hours (unless otherwise noted) and then moved to a new plate spread with OP50 or *S. aureus* and killing assays were performed as previously described incubating animals at 18°C [11]. A small number of worms were also treated with *smd-1* or *cir-1* dsRNA as controls for RNAi efficacy. For OP50 longevity experiments, nematodes were maintained on standard NGM plates with OP50 at 18°C. *P. aeruginosa* killing assays were similar to those described by Tan and colleagues using the “slow killing” method [48], [53] wherein L4 animals were added to NGM agar plates supplemented with 0.35% peptone and 0.1 mg/mL 5-fluorodeoxyuridine (FUDR), to prevent *C. elegans* self-fertilization. Unless otherwise noted, *S. aureus* and *P. aeruginosa* plates were prepared as described above.

25–50 L4-stage nematodes were added to each plate, and all assays were carried out in technical duplicate or triplicate. Plates were incubated at 18°C and scored for live and dead worms every 4–24 hours. A worm was considered dead when it did not respond to gentle plate tapping or touch with a platinum wire. Worms that died as a result of sticking to the wall of the plate, a spewing vulva, or bagging were censored from analysis. Statistical analyses for pathogen infection assays are described below.

### Survival data statistical analysis

Unless otherwise noted, each experiment was repeated at least twice. Each survival curve in the main text reflects data from at least two experiments and each experiment was analyzed statistically alone in Table S1. Curves were analyzed based on the TD50 (the time it takes 50% of nematodes to die) and nematode survival was calculated by the Kaplan-Meier method and survival differences were tested for significance by use of the log rank test (GraphPad Prism, version 4.0; GraphPad Software, Inc., San Diego, Calif., 2005). Significant difference were defined as *p*<0.0100. Individual experimental statistics for OP50- and pathogen-fed animals are included in the Table S1.

### Immunoblot

For immunoblots, nematodes were synchronized by bleaching and grown in M9 overnight. All strains were incubated at the same temperature prior to collection at the L4 stage. Worm pellets were lysed in TPER tissue protein extraction reagent (Thermo Scientific) supplemented with 1X Complete Mini protease inhibitors (Roche, 11836153001) and 1X PhosSTOP (Roche 04906845001) or 1 mM PMSF on ice by Cup Horn sonication at medium power on ice for 10 minutes (Misonix, Inc., Farmingddale, NY). Insoluble material was removed by centrifugation at 16,000×g for 10 min at 4°C and protein concentration was determined by NanoDrop spectrophotometer (Thermo Scientific). Similar amounts (∼250 micrograms) of total protein was added per lane after a 10 minute incubation at ∼90°C in NuPage LDS Sample Buffer (Invitrogen) supplemented by β-mercaptoethanol onto NuPage Bis-Tris gels (Invitrogen). Proteins were separated using MOPS buffer at 200 V after which time the proteins were transferred to nitrocellulose in NuPAGE transfer buffer at 100 V for 1.5 hours. To confirm efficient transfer, the membrane was stained with Ponceau S solution. The membrane was then was blocked for 45 min in Odyssey blocking buffer.

The *O-*GlcNAc specific antibody RL2 (Pierce, MA1-072) and rabbit anti-β-actin antibody (Abcam, ab1801) were diluted 1∶1000 in Odyssey blocking buffer with 0.1% Tween-20 and incubated with the membrane overnight at 4°C. The phospho-P38 MAPK specific (Cell Signaling, 4511) and anti-tubulin (Sigma Aldrich, T6074) antibodies were diluted 1∶1000 and 1∶1500, respectively, in 1X phosphate buffered saline with 0.1% Tween-20 (PBST) and incubated with the membrane overnight. Membranes were then washed three times in PBST. Secondary infrared 800- and 680-conjugated antibodies (Li-COR Biosciences, Lincoln, NE) at 1∶5000 dilution in Odyssey blocking buffer with 0.1% Tween-20 were incubated with membranes for 1 hour at RT in the dark. Finally, membranes were washed three times in PBST and imaged using the Odyssey Infrared Imaging System (LI-COR Biosciences, Lincoln, NE) according to the manufacturer’s instructions.

### Healthspan

Animals were synchronized and maintained at 18°C until the L4 larval stage at which point they were picked onto cdc-25.1 dsRNA-expressing *E. coli* HT115 or FUDR-containing *P. aerigunosa* plates. Animals on RNAi plates were then exposed to OP50 or *S. aureus* after 24 hours. Animals were maintained on the OP50 or pathogenic bacteria plates for 18 hours after which time, their pharyngeal pumping was assessed by observing the number of pharyngeal contractions during a 20 sec interval under a dissecting microscope. The backward movement of the terminal bulb of the pharynx was considered a contraction. The pumping rates for at least 10 nematodes per genotype were counted and analyzed. To determine the significance of pumping rate differences, one-way ANOVA analysis was used (GraphPad Prism, version 4.0; GraphPad Software, Inc., San Diego, Calif., 2005). Significant differences were defined as *p*<0.0100. Individual experimental statistics are included in Table S2.

### Brood size measurements

Synchronized N2 and mutant L4 worms were singly picked onto plates seeded with OP50, incubated at room temperature, and allowed to lay eggs. Adult animals were transferred daily to fresh plates until the end of the reproductive period. For experiments with *pmk-1(km25)* mutants, any parental animals that died within the first 48 hours were censored. As *bar-1* mutants have phenotypes including a shortened lifespan and bagging, upon bagging, parental worms were removed from the plate. The resulting progeny were allowed to develop for an additional 1–2 days at which time, the total number of laid progeny for each animal was counted. At least 29 animals were used for the analysis of each strain. To determine the significance of brood size differences, one-way ANOVA analysis was used (GraphPad Prism, version 4.0; GraphPad Software, Inc., San Diego, Calif., 2005). Significant differences were defined as *p*<0.0010. Individual experimental statistics are included in Table S3.

### Microscopy

Nematodes were examined using differential interference contrast microscopy with Nomarski optics and by confocal fluorescence microscopy using a Zeiss LSM700 microscope and established techniques [54]. Briefly, worms were incubated with pathogenic or non-pathogenic bacteria for 24 hours after which time they were moved to a plate devoid of bacteria for 15 min. Worms were then mounted on a ∼0.4 mm-thick pad of agar on slide in 0.1 M levamisole to immobilize the worms. At least 10 worms were assessed for each duplicate or triplicate experiment and a representative image is used in the text. Photoshop CS3 was used to balance images for brightness and contrast as well as to rotate the images for consistent anterior-posterior presentation. Fluorescent images were manipulated identically for brightness and contrast.

### C. elegans growth and collection for microarray analysis using Affymetrix GeneChips

In triplicate, L4 N2, *ogt-1(ok1474)*, *oga-1(ok1207)*, and *pmk-1(km25)* strains were exposed to OP50, *S. aureus*, or *P. aeruginosa* for 6 hours. *E. coli* OP50 was cultured as described above on 2% agarose-topped NGM plates. The *S. aureus* NCTC8325 strain was cultured overnight at 37°C with aeration in tryptic soy broth containing 10 µg/mL nalidixic acid. 50 µL of *S. aureus* was seeded directly onto 2% agarose-topped TSA plates at 37°C for 24 hours and RT for 24 hours. *P. aeruginosa* was cultured in King’s Broth containing 100 µg/mL rifampicin. 200 µL of *P. aeruginosa* was seeded directly onto 2% agarose-topped 0.35% peptone NGM plates containing the appropriate antibiotics and incubated overnight at 37°C after which they were incubated at RT for 24 hours.

Synchronized L4 nematodes were washed from NGM plates, counted, and purged by gently rocking the animals for 15 min at room temperature in M9. After this time, nematodes were divided equally and subjected to 6 hours of OP50, *S. aureus*, or *P. aeruginosa* exposure at 18°C. Nematodes were removed from bacterial plates, washed extensively, and purged for 15 min to remove any remaining bacteria. Collected worms were centrifuged in M9 and all supernatant removed. Samples were flash-frozen and stored at −80°C until RNA preparation.

### Microarray target preparation and hybridization for Affymetrix Gene Chips

Performed in triplicate, RNA was isolated from frozen nematode samples. Briefly, pellets were resuspended in RLT lysis buffer and added to a stainless steel microvial (Biospec Products Inc. #2007) containing a stainless steel ball bearing. Worms were lysed with a bead beater for 10 seconds of shaking followed by 5 min incubation on ice, repeated six times. Samples were moved and centrifuged at 4,000×g for 5 min at 4°C and the supernatant moved to a new tube after which they were further processed according to the RNeasy Mini Kit (Qiagen #74104).

Genotypes were confirmed for all samples following the above RNA isolation protocol. 1.5 µg RNA was treated with DNAse for 15 min at RT and the reaction stopped by the addition of 25 mM EDTA and incubation at 65°C for 10 min. Resulting samples were utilized to produce cDNA using the SuperScript-III kit (Invitrogen #18080-044), random primers (Promega C118A), DNTPs (Invitrogen 18427-013), and RNAseOUT (Invitrogen 10777-019). Following cDNA synthesis, Real-time PCR was performed using SYBR Green to qualitatively determine the presence or absence of a specific allele with the following reaction conditions: 1.25 µL cDNA, 10.25 µL water, 0.5 µL forward and reverse primers (10 µM working concentration), 12.5 µL 2X SYBR green mixture. Primers are listed below. Ct values below 22 indicated the presence of a transcript while Ct values above 34 indicated no transcript was present.

The following primers were used to confirm sample genotypes: control *oga-1* forward (acaagcaccaaacaaggcaat), control *oga-1* reverse (tgtgggcatcaaacgcata), deletion *oga-1* forward (ttggctcgagtgggaagtgt), deletion *oga-1* reverse (gcatgcaagttgtcccagataa), control *ogt-1* forward (tgttctgaaagaggccaggatt), control *ogt-1* reverse (cagattcaaggctcggagataag), deletion *ogt-1* forward (tcacatgcggctcggatt), deletion *ogt-1* reverse (gaaccttatcgaagcacaacga), *act-1* forward (tgcgacattgatatccgtaagg), *act-1* reverse (ggtggttcctccggaaagaa), control *pmk-1* forward (ttgttccctggatctgatcaca), control *pmk-1* reverse (tttgaaatcacggcgagtca), deletion *pmk-1* forward (cgcgtcgcaatcaaaaaatt), deletion *pmk-1* reverse (ctcgatatcatttacattctcatttgg).

After correct genotypes were confirmed, RNA samples were prepared for microarray analysis. RNA quality was tested using bioanalyzer (RNA Nano assay in the Expert 2100 software, Agilent Technologies, CA) and RNA Integrity Numbers (RIN) were above 9.7 for all samples. 100 ng of total RNA from each sample was amplified to generate cDNA using NUGEN Applause 3′Amp system (NuGEN Technologies, CA), according to the manufacturer’s instructions. 4 µg of cDNA from each sample was fragmented and biotinylated using Encore Biotin module (NUGEN Technologies, CA). Resultant sample was mixed with hybridization reagents (Affymetrix, Inc.) and injected into Affymetrix full-genome GeneChips for *C. elegans* and incubated in hybridization oven rotating 60 rpm at 45°C for 18±2 h. Arrays were processed using Affymetrix 450 Fluidic station using wash and stain kit (Affymetrix, Inc.). Chips were scanned using Affymetrix GeneChip scanner 3000 operated by Gene Chip Operating Software, version 1. 4 (GCOS 1.4) and generated .CEL, .CHP and .RPT files for statistical analysis. To access the efficiency of cDNA synthesis PolyA controls (dap, lys, phe, thr- Affymetrix Inc.) were spiked to the samples and hybridization controls (bioB, bioD, bioC and Cre, Affymetrix Inc.) were added to monitor labeling efficiency according to the manufacturer’s instructions. These procedures were performed by the NIDDK Genomics Core Laboratory. Microarray signals were normalized using the RMA algorithm. The significantly expressed genes were selected based on ANOVA analysis by Partek Genomics Suite software (Partek, St. Charles, MO, USA). Genes with a *p*-value of <0.05 and a 2-fold or greater fold change were considered differentially expressed.

### Quantitative RT-PCR (qRT-PCR) analysis

Transcript levels of transgenes in N2, *ogt-1(ok1474)*, *oga-1(ok1207)*, *pmk-1(km25)*, and *ogt-1(ok1474)*; *pmk-1(km25)* strains were measured by RT-PCR. RT-PCR was used to confirm the pathogen-induced changes in gene expression for several genes. Total RNA was obtained from the same samples used for microarray analysis. RNA was reverse transcribed as described above and cDNA was subjected to RT-qPCR analysis using SYBR green detection on the CFX96 from BioRad. StellARray qPCR Arrays, including primer sets, were obtained from Bar Harbor Bio Technologies, Inc. Results are the average of three biological replicates normalized to the geometric mean of two control genes (*snb-1* and *gpd-3*). Changes were determined by normalizing samples to a control sample. Error bars represent standard deviation.

## Supporting Information

Figure S1 **Deletion of** ***ogt-1*** **and** ***oga-1*** **influence O-GlcNAc levels in mutant** ***C. elegans*** **strains.** The *ogt-1* (A) and *oga-1* (B) gene schematics based on Wormbase release WS239 with exons shown as colored boxes and introns as black lines. The *ogt-1* gene is found on chromosome III: −0.78 +/− 0.001 cM and *oga-1* is found on X: 1.07 +/− 0.015 cM. Brackets indicate four deletions: *ogt-1(ok430), ogt-1(ok1474), oga-1(ok1207),* and *oga-1(tm3642)*. (C) Representative anti-O-GlcNAc immunoblot with anti-β-actin as loading control demonstrates that strains lacking catalytically active OGT-1 (*ogt-1(ok430) and (ok1474)*) exhibit absence of O-GlcNAc signal while strains lacking OGA-1 activity (*oga-1(ok1207) and (tm3642)*) show an increase in O-GlcNAc signal.
(TIF)

Figure S2 **Lifespan values for each genotype exposed to OP50 after cdc25.1 RNAi treatment representing at least two independent biological replicates.** (A) and (B) Each circle represents the median lifespan (in hours after the L4 transfer to OP50) for an individual assay done in technical duplicate or triplicate. This summary of lifespan data demonstrates that animals with O-GlcNAc cycling and *pmk-1* mutant backgrounds are generally healthy while those with *bar-1* mutant backgrounds have a decreased lifespan. Assays were performed with OP50 under conditions described in *Materials and Methods*. *n*≧162 animals.
(TIF)

Figure S3 **OP50** ***E. coli*** **-fed nematodes are generally healthy while pathogen-fed worms demonstrate a decrease in overall health.** (A) Representative images of OP50-GFP fed animals that exhibit no intestinal accumulation of bacteria after feeding for 18 hours as evidenced by a dearth GFP-labeled bacteria past the pharyngeal grinder. (B) *ogt-1, oga-1,* and *pmk-1(km25)* have brood sizes similar to N2 while (C) *bar-1(ga80)* mutants show a decrease in brood size. Statistical differences are compared to N2. *n*≧29. Statistical significance defined as: *p*≤0.01. ****p<0.0001 and ***p≤0.001 with statistical data found in Table S3. (D) *P. aeruginosa*-GFP fed worms exhibit variable levels of bacterial accumulation after 18 hours of exposure. (E) *S. aureus*-GFP fed worms exhibit intestinal accumulation of bacteria after 18 hours of exposure and a distension of the intestinal lumen. *n*≧10 worms for each experiment done in at least duplicate.
(TIF)

Figure S4 **Levels of activated, phosphorylated PMK-1 remain similar to N2 in O-GlcNAc cycling mutants.** Representative immunoblots with anti phospho-p38 MAPK antibody and anti-tubulin (A) or anti-β-actin as loading control (B). Strains lacking either *pmk-1* (A) or *sek-1* (B) (the kinase required for *pmk-1* phosphorylation) show complete loss of signal for active, phosphorylated PMK-1. Graph indicates relative levels of signal normalized to N2 with error bars representing range.
(TIF)

Figure S5 **Genes are differentially induced in N2 and mutant worms upon** ***S. aureus*** **exposure.** RT-qPCR analysis shows the induction or repression of selected genes: *fmo-2*, *clec-60*, *cnc-7*, *nspe-5*, *abf-2*, *lipl-2*, *ilys-2*, *irg-2*, Y75B8A.23, Y41C4A.11, and *pgp-1*. Data are the relative levels of expression of genes differentially regulated in mutant backgrounds compared to N2 on OP50 and those deregulated within an individual mutant upon pathogen exposure. Data in the heat map (A) represent the means of three biological replicates and are normalized to the geometric mean of two housekeeping genes (*snb-1* and *gpd-3*) (B). N2, *ogt-1(ok1474)*, *oga-1(ok1207)*, *pmk-1(km25)*, and *ogt-1(ok1474); pmk-1(km25)* strains were used.
(TIF)

Figure S6 ***S. aureus*** **exposed animals do not undergo starvation response.** 18 fasting response genes were identified by qRT-PCR by Van Gilst and colleagues [31]. Maximum fold repression in starved L4 ranged from −1.6 (*ech-1*) to −55.0 (*lbp-8*) (red triangle) while maximum fold induction in starved L4 ranged from 2.8 (*fat-2*) to 39.8 (*acs-2*) (green triangle). The transcriptional responses in N2, *ogt-1(ok1474),* and *oga-1(ok1207)* animals fed *S. aureus* were compared to the same genotypes fed OP50 by microarray and changes were generally negligible. Bars highlighted in green indicate increases in gene expression while bars highlighted in red indicate decreases in gene expression over 2-fold. Orange bars represent gene expression changes <2-fold.
(TIF)

Figure S7 **Survival of** ***bar-1*** **single and double mutants after 48 hr cdc25.1 RNAi exposure.** *bar-1(ga80)* single mutants, *ogt-1(ok430); bar-1(ga80)*, and *oga-1(tm3642) bar-1(ga80)* double mutants have similar survivals. N2 appears to have a lengthened survival likely due to persistent cdc25.1 RNAi presence. Killing assays were performed with *S. aureus* under conditions described in *Materials and Methods* and are represented here by comprehensive plots. *n*≧154. Statistics for all experiments are available in Table S1.
(TIF)

Table S1 **Lifespan statistics.**
(PDF)

Table S2 **Pumping rate statistics.**
(PDF)

Table S3 **Broodsize statistics.**
(PDF)

Table S4 **Genes enriched in** ***ogt-1, oga-1,*** **and** ***pmk-1*** **mutants by microarray.**
(PDF)

Dataset S1 **OP50,** ***S. aureus*** **, and** ***P. aeruginosa*** **Microarray ANOVA expression analysis.**
(XLSX)

## References

[1] Engelmann I, Pujol N (2010) Innate immunity in C. elegans. editors. Invertebrate Immunity. Springer. 105–121. 10.1007/978-1-4419-8059-5_6 21528695

[2] Gravato-Nobre MJ, Hodgkin J (2005) Caenorhabditis elegans as a model for innate immunity to pathogens. Cellular microbiology 7:741–751. 15888078 10.1111/j.1462-5822.2005.00523.x

[3] Zachara NE, Hart GW (2004) O-GlcNAc a sensor of cellular state: the role of nucleocytoplasmic glycosylation in modulating cellular function in response to nutrition and stress. Biochimica et Biophysica Acta (BBA)-General Subjects 1673:13–28. 15238246 10.1016/j.bbagen.2004.03.016

[4] Bond MR, Hanover JA (2013) O-GlcNAc Cycling: A Link Between Metabolism and Chronic Disease. Annual review of nutrition 33:205–229. 10.1146/annurev-nutr-071812-161240 PMC10483992 23642195

[5] Love DC, Ghosh S, Mondoux MA, Fukushige T, Wang P, et al (2010) Dynamic O-GlcNAc cycling at promoters of Caenorhabditis elegans genes regulating longevity, stress, and immunity. Proceedings of the National Academy of Sciences 107:7413–7418. 10.1073/pnas.0911857107 PMC2867743 20368426

[6] Byrne AB, Weirauch MT, Wong V, Koeva M, Dixon SJ, et al (2007) A global analysis of genetic interactions in Caenorhabditis elegans. J Biol 6:8. 17897480 10.1186/jbiol58 PMC2373897

[7] Ha JR, Hao L, Venkateswaran G, Huang YH, Garcia E, et al (2013) beta-catenin is O-GlcNAc Glycosylated at Serine 23: Implications for beta-catenin’s Subcellular Localization and Transactivator Function. Exp Cell Res 321:153–166. 24342833 10.1016/j.yexcr.2013.11.021

[8] Olivier-Van Stichelen S, Drougat L, Dehennaut V, El Yazidi-Belkoura I, Guinez C, et al (2012) Serum-stimulated cell cycle entry promotes ncOGT synthesis required for cyclin D expression. Oncogenesis 1:e36. 23552487 10.1038/oncsis.2012.36 PMC3545199

[9] Sayat R, Leber B, Grubac V, Wiltshire L, Persad S (2008) O-GlcNAc-glycosylation of β-catenin regulates its nuclear localization and transcriptional activity. Experimental cell research 314:2774–2787. 18586027 10.1016/j.yexcr.2008.05.017

[10] Cheung WD, Hart GW (2008) AMP-activated protein kinase and p38 MAPK activate O-GlcNAcylation of neuronal proteins during glucose deprivation. Journal of Biological Chemistry 283:13009–13020. 18353774 10.1074/jbc.M801222200 PMC2435304

[11] Irazoqui JE, Troemel ER, Feinbaum RL, Luhachack LG, Cezairliyan BO, et al (2010) Distinct pathogenesis and host responses during infection of C. elegans by P. aeruginosa and S. aureus. PLoS pathogens 6:e1000982. 20617181 10.1371/journal.ppat.1000982 PMC2895663

[12] Wellen KE, Thompson CB (2012) A two-way street: reciprocal regulation of metabolism and signalling. Nature reviews Molecular cell biology 13:270–276. 22395772 10.1038/nrm3305

[13] Evans EA, Chen WC, Tan MW (2008) The DAF-2 insulin-like signaling pathway independently regulates aging and immunity in C. elegans. Aging Cell 7:879–893. 18782349 10.1111/j.1474-9726.2008.00435.x PMC2630471

[14] Garsin DA, Villanueva JM, Begun J, Kim DH, Sifri CD, et al (2003) Long-lived C. elegans daf-2 mutants are resistant to bacterial pathogens. Science 300:1921. 12817143 10.1126/science.1080147

[15] Irazoqui JE, Ng A, Xavier RJ, Ausubel FM (2008) Role for β-catenin and HOX transcription factors in Caenorhabditis elegans and mammalian host epithelial-pathogen interactions. Proceedings of the National Academy of Sciences 105:17469–17474. 10.1073/pnas.0809527105 PMC2582251 18981407

[16] Mondoux MA, Love DC, Ghosh SK, Fukushige T, Bond M, et al (2011) O-linked-N-acetylglucosamine cycling and insulin signaling are required for the glucose stress response in Caenorhabditis elegans. Genetics 188:369–382. 21441213 10.1534/genetics.111.126490 PMC3122314

[17] Rahman MM, Stuchlick O, El-Karim EG, Stuart R, Kipreos ET, et al (2010) Intracellular protein glycosylation modulates insulin mediated lifespan in C. elegans. Aging (Albany NY) 2:678–690. 20952811 10.18632/aging.100208 PMC2993798

[18] Huang C, Xiong C, Kornfeld K (2004) Measurements of age-related changes of physiological processes that predict lifespan of Caenorhabditis elegans. Proc Natl Acad Sci U S A 101:8084–8089. 15141086 10.1073/pnas.0400848101 PMC419561

[19] Kong C, Tan MW, Nathan S (2014) Orthosiphon stamineus protects Caenorhabditis elegans against Staphylococcus aureus infection through immunomodulation. Biol Open 3:644–655. 24972867 10.1242/bio.20148334 PMC4154301

[20] Forsythe ME, Love DC, Lazarus BD, Kim EJ, Prinz WA, et al (2006) Caenorhabditis elegans ortholog of a diabetes susceptibility locus: oga-1 (O-GlcNAcase) knockout impacts O-GlcNAc cycling, metabolism, and dauer. Proceedings of the National Academy of Sciences 103:11952–11957. 10.1073/pnas.0601931103 PMC1567679 16882729

[21] Kim DH, Feinbaum R, Alloing G, Emerson FE, Garsin DA, et al (2002) A conserved p38 MAP kinase pathway in Caenorhabditis elegans innate immunity. Science 297:623–626. 12142542 10.1126/science.1073759

[22] Shivers RP, Pagano DJ, Kooistra T, Richardson CE, Reddy KC, et al (2010) Phosphorylation of the conserved transcription factor ATF-7 by PMK-1 p38 MAPK regulates innate immunity in Caenorhabditis elegans. PLoS genetics 6:e1000892. 20369020 10.1371/journal.pgen.1000892 PMC2848548

[23] Troemel ER, Chu SW, Reinke V, Lee SS, Ausubel FM, et al (2006) p38 MAPK regulates expression of immune response genes and contributes to longevity in C. elegans. PLoS genetics 2:e183. 17096597 10.1371/journal.pgen.0020183 PMC1635533

[24] Huang Da W, Sherman BT, Lempicki RA (2008) Systematic and integrative analysis of large gene lists using DAVID bioinformatics resources. Nature protocols 4:44–57. 10.1038/nprot.2008.211 19131956

[25] Bogaerts A, Temmerman L, Boerjan B, Husson SJ, Schoofs L, et al (2010) A differential proteomics study of Caenorhabditis elegans infected with Aeromonas hydrophila. Dev Comp Immunol 34:690–698. 20149819 10.1016/j.dci.2010.02.003

[26] Nandakumar M, Tan MW (2008) Gamma-linolenic and stearidonic acids are required for basal immunity in Caenorhabditis elegans through their effects on p38 MAP kinase activity. PLoS Genet 4:e1000273. 19023415 10.1371/journal.pgen.1000273 PMC2581601

[27] Sahu SN, Lewis J, Patel I, Bozdag S, Lee JH, et al (2012) Genomic analysis of immune response against Vibrio cholerae hemolysin in Caenorhabditis elegans. PLoS One 7:e38200. 22675448 10.1371/journal.pone.0038200 PMC3364981

[28] Simonsen KT, Gallego SF, Faergeman NJ, Kallipolitis BH (2012) Strength in numbers: “Omics” studies of C. elegans innate immunity. Virulence 3:477–484. 23076279 10.4161/viru.21906 PMC3524146

[29] Dunbar TL, Yan Z, Balla KM, Smelkinson MG, Troemel ER (2012) C. elegans detects pathogen-induced translational inhibition to activate immune signaling. Cell Host Microbe 11:375–386. 22520465 10.1016/j.chom.2012.02.008 PMC3334869

[30] Alper S, McBride SJ, Lackford B, Freedman JH, Schwartz DA (2007) Specificity and complexity of the Caenorhabditis elegans innate immune response. Mol Cell Biol 27:5544–5553. 17526726 10.1128/MCB.02070-06 PMC1952075

[31] Van Gilst MR, Hadjivassiliou H, Yamamoto KR (2005) A Caenorhabditis elegans nutrient response system partially dependent on nuclear receptor NHR-49. Proc Natl Acad Sci U S A 102:13496–13501. 16157872 10.1073/pnas.0506234102 PMC1201344

[32] Essers MA, de Vries-Smits LM, Barker N, Polderman PE, Burgering BM, et al (2005) Functional interaction between beta-catenin and FOXO in oxidative stress signaling. Science 308:1181–1184. 15905404 10.1126/science.1109083

[33] Eisenmann DM, Maloof JN, Simske JS, Kenyon C, Kim SK (1998) The beta-catenin homolog BAR-1 and LET-60 Ras coordinately regulate the Hox gene lin-39 during Caenorhabditis elegans vulval development. Development 125:3667–3680. 9716532 10.1242/dev.125.18.3667

[34] Natarajan L, Witwer NE, Eisenmann DM (2001) The divergent Caenorhabditis elegans beta-catenin proteins BAR-1, WRM-1 and HMP-2 make distinct protein interactions but retain functional redundancy in vivo. Genetics 159:159–172. 11560894 10.1093/genetics/159.1.159 PMC1461775

[35] Li J, He J, Yu C (2012) Chitosan oligosaccharide inhibits LPS-induced apoptosis of vascular endothelial cells through the BKCa channel and the p38 signaling pathway. International Journal of Molecular Medicine 30:157–164. 22469656 10.3892/ijmm.2012.954

[36] Pathak S, Borodkin VS, Albarbarawi O, Campbell DG, Ibrahim A, et al (2012) O-GlcNAcylation of TAB1 modulates TAK1-mediated cytokine release. The EMBO journal 31:1394–1404. 22307082 10.1038/emboj.2012.8 PMC3321193

[37] Sifri CD, Begun J, Ausubel FM, Calderwood SB (2003) Caenorhabditis elegans as a model host for Staphylococcus aureus pathogenesis. Infection and immunity 71:2208–2217. 12654843 10.1128/IAI.71.4.2208-2217.2003 PMC152095

[38] Iyer SP, Hart GW (2003) Roles of the tetratricopeptide repeat domain in O-GlcNAc transferase targeting and protein substrate specificity. J Biol Chem 278:24608–24616. 12724313 10.1074/jbc.M300036200

[39] Sifri CD, Begun J, Ausubel FM (2005) The worm has turned–microbial virulence modeled in Caenorhabditis elegans. Trends Microbiol 13:119–127. 15737730 10.1016/j.tim.2005.01.003

[40] Lee J, Kim KY, Lee J, Paik YK (2010) Regulation of Dauer formation by O-GlcNAcylation in Caenorhabditis elegans. J Biol Chem 285:2930–2939. 19940149 10.1074/jbc.M109.022665 PMC2823417

[41] Inoue H, Hisamoto N, An JH, Oliveira RP, Nishida E, et al (2005) The C. elegans p38 MAPK pathway regulates nuclear localization of the transcription factor SKN-1 in oxidative stress response. Genes & development 19:2278–2283. 16166371 10.1101/gad.1324805 PMC1240035

[42] Pearson JC, Lemons D, McGinnis W (2005) Modulating Hox gene functions during animal body patterning. Nat Rev Genet 6:893–904. 16341070 10.1038/nrg1726

[43] Wang BB, Muller-Immergluck MM, Austin J, Robinson NT, Chisholm A, et al (1993) A homeotic gene cluster patterns the anteroposterior body axis of C. elegans. Cell 74:29–42. 8101474 10.1016/0092-8674(93)90292-x

[44] Kneass ZT, Marchase RB (2004) Neutrophils exhibit rapid agonist-induced increases in protein-associated O-GlcNAc. J Biol Chem 279:45759–45765. 15322139 10.1074/jbc.M407911200

[45] Golks A, Tran TT, Goetschy JF, Guerini D (2007) Requirement for O-linked N-acetylglucosaminyltransferase in lymphocytes activation. EMBO J 26:4368–4379. 17882263 10.1038/sj.emboj.7601845 PMC2034663

[46] Pattee PA (1990) Genetic and physical mapping of the chromosome of Staphylococcus aureus NCTC 8325. Washington, DC: American Society for Microbiology.

[47] Cheung AL, Nast CC, Bayer AS (1998) Selective activation of sar promoters with the use of green fluorescent protein transcriptional fusions as the detection system in the rabbit endocarditis model. Infect Immun 66:5988–5993. 9826382 10.1128/iai.66.12.5988-5993.1998 PMC108758

[48] Tan M-W, Mahajan-Miklos S, Ausubel FM (1999) Killing of Caenorhabditis elegans by Pseudomonas aeruginosa used to model mammalian bacterial pathogenesis. Proceedings of the National Academy of Sciences 96:715–720. 10.1073/pnas.96.2.715 PMC15202 9892699

[49] Brenner S (1974) The genetics of Caenorhabditis elegans. Genetics 77:71–94. 4366476 10.1093/genetics/77.1.71 PMC1213120

[50] Lewis JA, Fleming JT (1995) Basic culture methods. Methods Cell Biol 48:3–29. 8531730

[51] Eisenmann DM, Maloof JN, Simske JS, Kenyon C, Kim SK (1998) The beta-catenin homolog BAR-1 and LET-60 Ras coordinately regulate the Hox gene lin-39 during Caenorhabditis elegans vulval development. Development 125:3667–3680. 9716532 10.1242/dev.125.18.3667

[52] Gleason JE, Korswagen HC, Eisenmann DM (2002) Activation of Wnt signaling bypasses the requirement for RTK/Ras signaling during C. elegans vulval induction. Genes & development 16:1281–1290. 12023306 10.1101/gad.981602 PMC186276

[53] Mahajan-Miklos S, Tan MW, Rahme LG, Ausubel FM (1999) Molecular mechanisms of bacterial virulence elucidated using a Pseudomonas aeruginosa-Caenorhabditis elegans pathogenesis model. Cell 96:47–56. 9989496 10.1016/s0092-8674(00)80958-7

[54] Sulston J, Hodgkin J (1988) The Nematode Caenorhabditis elegans (Wood, WB, ed.) 587–606.

[55] Pukkila-Worley R, Feinbaum R, Kirienko NV, Larkins-Ford J, Conery AL, et al (2012) Stimulation of host immune defenses by a small molecule protects C. elegans from bacterial infection. PLoS Genet 8:e1002733. 22719261 10.1371/journal.pgen.1002733 PMC3375230

[56] Zugasti O, Ewbank JJ (2009) Neuroimmune regulation of antimicrobial peptide expression by a noncanonical TGF-beta signaling pathway in Caenorhabditis elegans epidermis. Nat Immunol 10:249–256. 19198592 10.1038/ni.1700

[57] Studencka M, Wesolowski R, Opitz L, Salinas-Riester G, Wisniewski JR, et al (2012) Transcriptional repression of Hox genes by C. elegans HP1/HPL and H1/HIS-24. PLoS Genet 8:e1002940. 23028351 10.1371/journal.pgen.1002940 PMC3441639

[58] Aballay A, Drenkard E, Hilbun LR, Ausubel FM (2003) Caenorhabditis elegans innate immune response triggered by Salmonella enterica requires intact LPS and is mediated by a MAPK signaling pathway. Curr Biol 13:47–52. 12526744 10.1016/s0960-9822(02)01396-9

[59] Couillault C, Pujol N, Reboul J, Sabatier L, Guichou JF, et al (2004) TLR-independent control of innate immunity in Caenorhabditis elegans by the TIR domain adaptor protein TIR-1, an ortholog of human SARM. Nat Immunol 5:488–494. 15048112 10.1038/ni1060

[60] Cui Y, McBride SJ, Boyd WA, Alper S, Freedman JH (2007) Toxicogenomic analysis of Caenorhabditis elegans reveals novel genes and pathways involved in the resistance to cadmium toxicity. Genome Biol 8:R122. 17592649 10.1186/gb-2007-8-6-r122 PMC2394766

[61] Engelmann I, Griffon A, Tichit L, Montanana-Sanchis F, Wang G, et al (2011) A comprehensive analysis of gene expression changes provoked by bacterial and fungal infection in C. elegans. PLoS One 6:e19055. 21602919 10.1371/journal.pone.0019055 PMC3094335

[62] Sun J, Liu Y, Aballay A (2012) Organismal regulation of XBP-1-mediated unfolded protein response during development and immune activation. EMBO Rep 13:855–860. 22791024 10.1038/embor.2012.100 PMC3432796

