# Supplementary figures and images for: Conserved Nutrient Sensor O-GlcNAc Transferase Is Integral to C. elegans Pathogen-Specific Immunity
Source: PLoS One. 2014 Dec 4;9(12):e113231. doi: 10.1371/journal.pone.0113231 (PMC4256294; doi:10.1371/journal.pone.0113231)

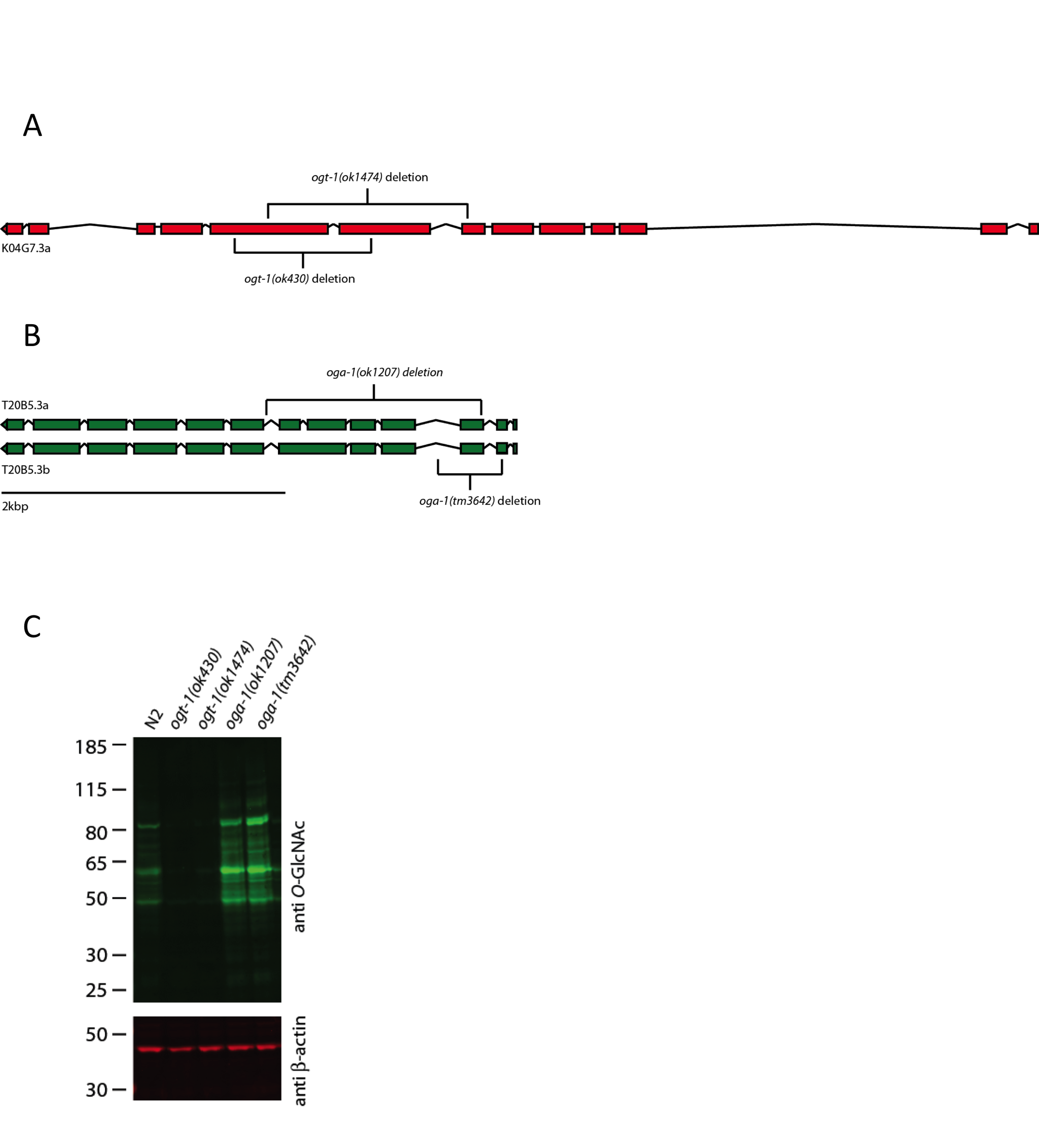

Supplement: Figure S1 — Deletion of ogt-1 and oga-1 influence O-GlcNAc levels in mutant C. elegans strains. The ogt-1 (A) and oga-1 (B) gene schematics based on Wormbase release WS239 with exons shown as colored boxes and introns as black lines. The ogt-1 gene is found on chromosome III: −0.78 +/− 0.001 cM and oga-1 is found on X: 1.07 +/− 0.015 cM. Brackets indicate four deletions: ogt-1(ok430), ogt-1(ok1474), oga-1(ok1207), and oga-1(tm3642). (C) Representative anti-O-GlcNAc immunoblot with anti-β-actin as loading control demonstrates that strains lacking catalytically active OGT-1 (ogt-1(ok430) and (ok1474)) exhibit absence of O-GlcNAc signal while strains lacking OGA-1 activity (oga-1(ok1207) and (tm3642)) show an increase in O-GlcNAc signal. (TIF) [file pone.0113231.s001.tif]

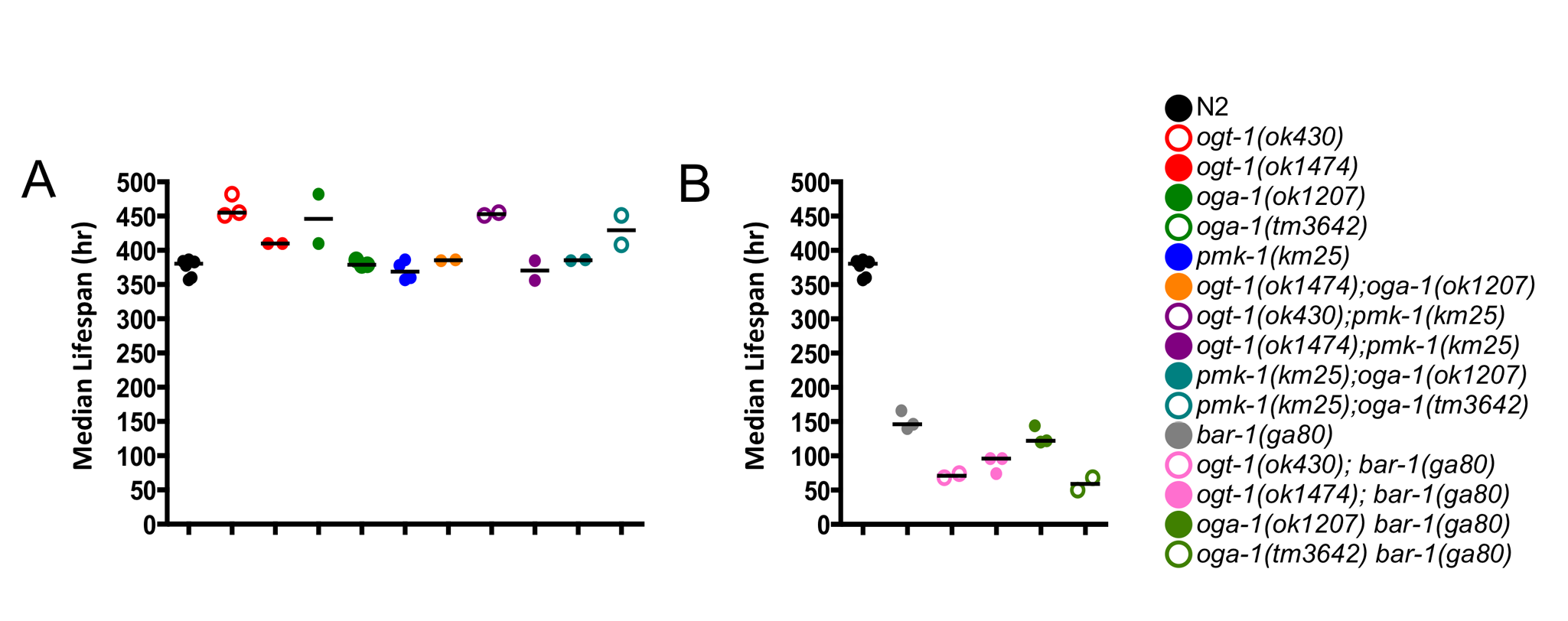

Supplement: Figure S2 — Lifespan values for each genotype exposed to OP50 after cdc25.1 RNAi treatment representing at least two independent biological replicates. (A) and (B) Each circle represents the median lifespan (in hours after the L4 transfer to OP50) for an individual assay done in technical duplicate or triplicate. This summary of lifespan data demonstrates that animals with O-GlcNAc cycling and pmk-1 mutant backgrounds are generally healthy while those with bar-1 mutant backgrounds have a decreased lifespan. Assays were performed with OP50 under conditions described in Materials and Methods . n≧162 animals. (TIF) [file pone.0113231.s002.tif]

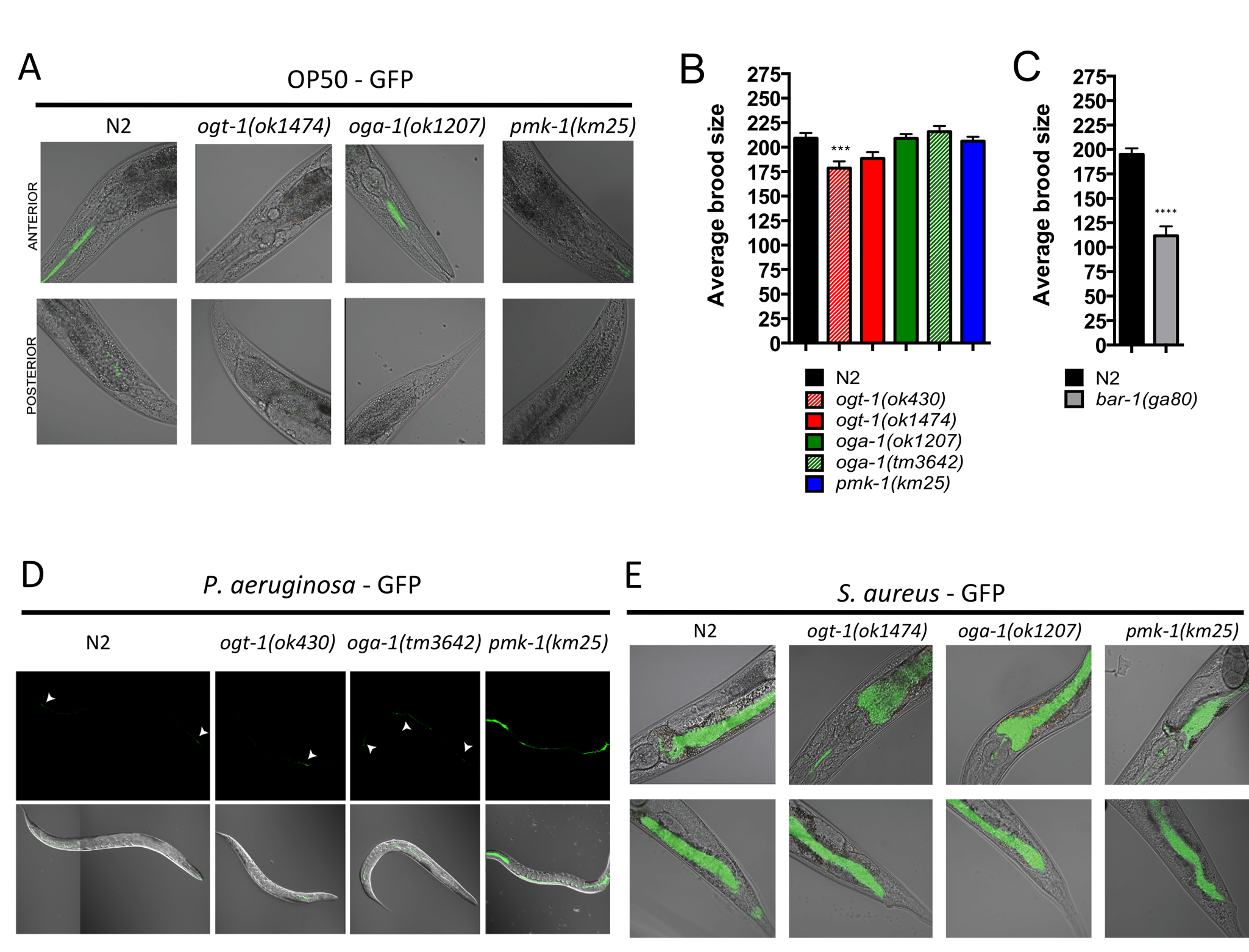

Supplement: Figure S3 — OP50 E. coli -fed nematodes are generally healthy while pathogen-fed worms demonstrate a decrease in overall health. (A) Representative images of OP50-GFP fed animals that exhibit no intestinal accumulation of bacteria after feeding for 18 hours as evidenced by a dearth GFP-labeled bacteria past the pharyngeal grinder. (B) ogt-1, oga-1, and pmk-1(km25) have brood sizes similar to N2 while (C) bar-1(ga80) mutants show a decrease in brood size. Statistical differences are compared to N2. n≧29. Statistical significance defined as: p≤0.01. ****p<0.0001 and ***p≤0.001 with statistical data found in Table S3. (D) P. aeruginosa-GFP fed worms exhibit variable levels of bacterial accumulation after 18 hours of exposure. (E) S. aureus-GFP fed worms exhibit intestinal accumulation of bacteria after 18 hours of exposure and a distension of the intestinal lumen. n≧10 worms for each experiment done in at least duplicate. (TIF) [file pone.0113231.s003.tif]

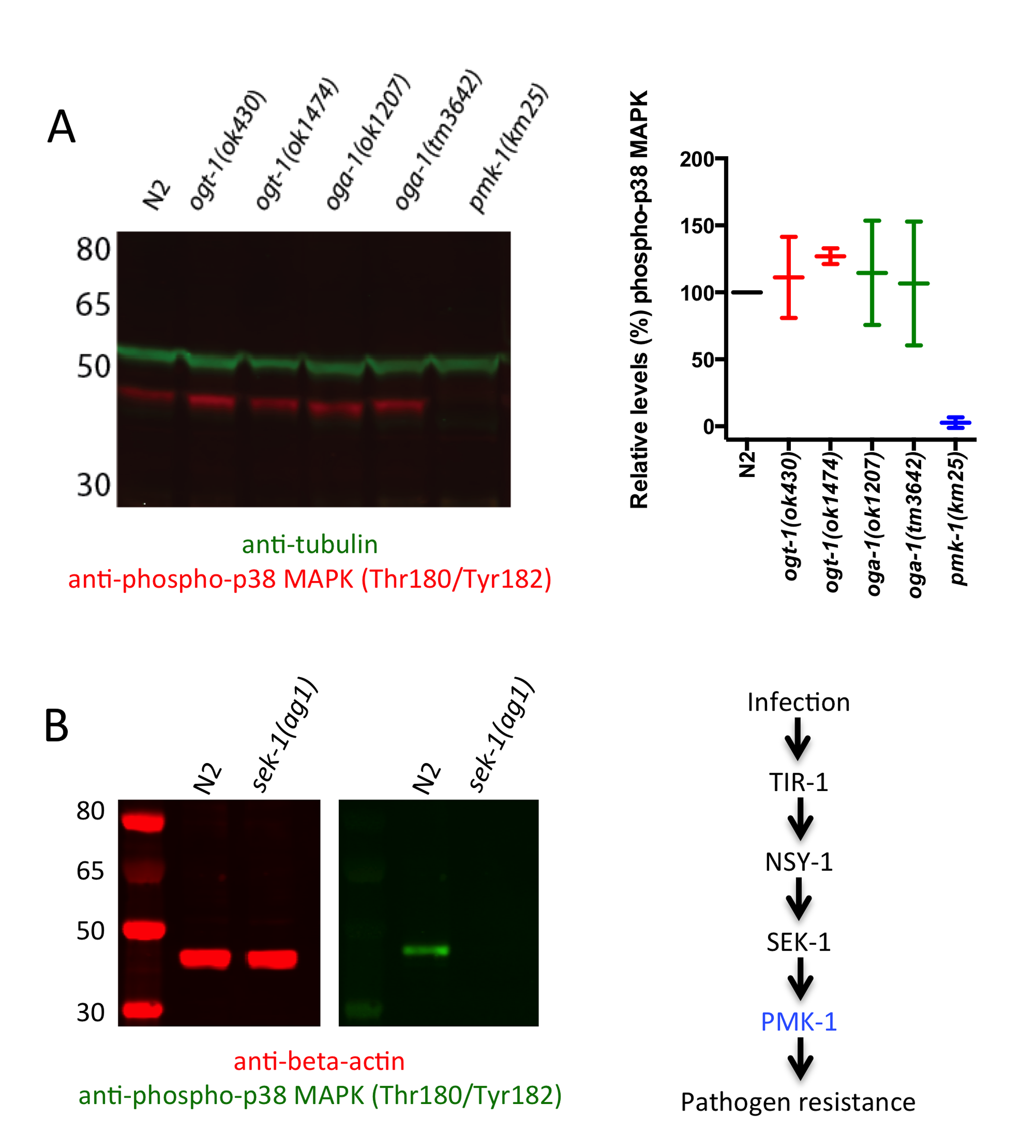

Supplement: Figure S4 — Levels of activated, phosphorylated PMK-1 remain similar to N2 in O-GlcNAc cycling mutants. Representative immunoblots with anti phospho-p38 MAPK antibody and anti-tubulin (A) or anti-β-actin as loading control (B). Strains lacking either pmk-1 (A) or sek-1 (B) (the kinase required for pmk-1 phosphorylation) show complete loss of signal for active, phosphorylated PMK-1. Graph indicates relative levels of signal normalized to N2 with error bars representing range. (TIF) [file pone.0113231.s004.tif]

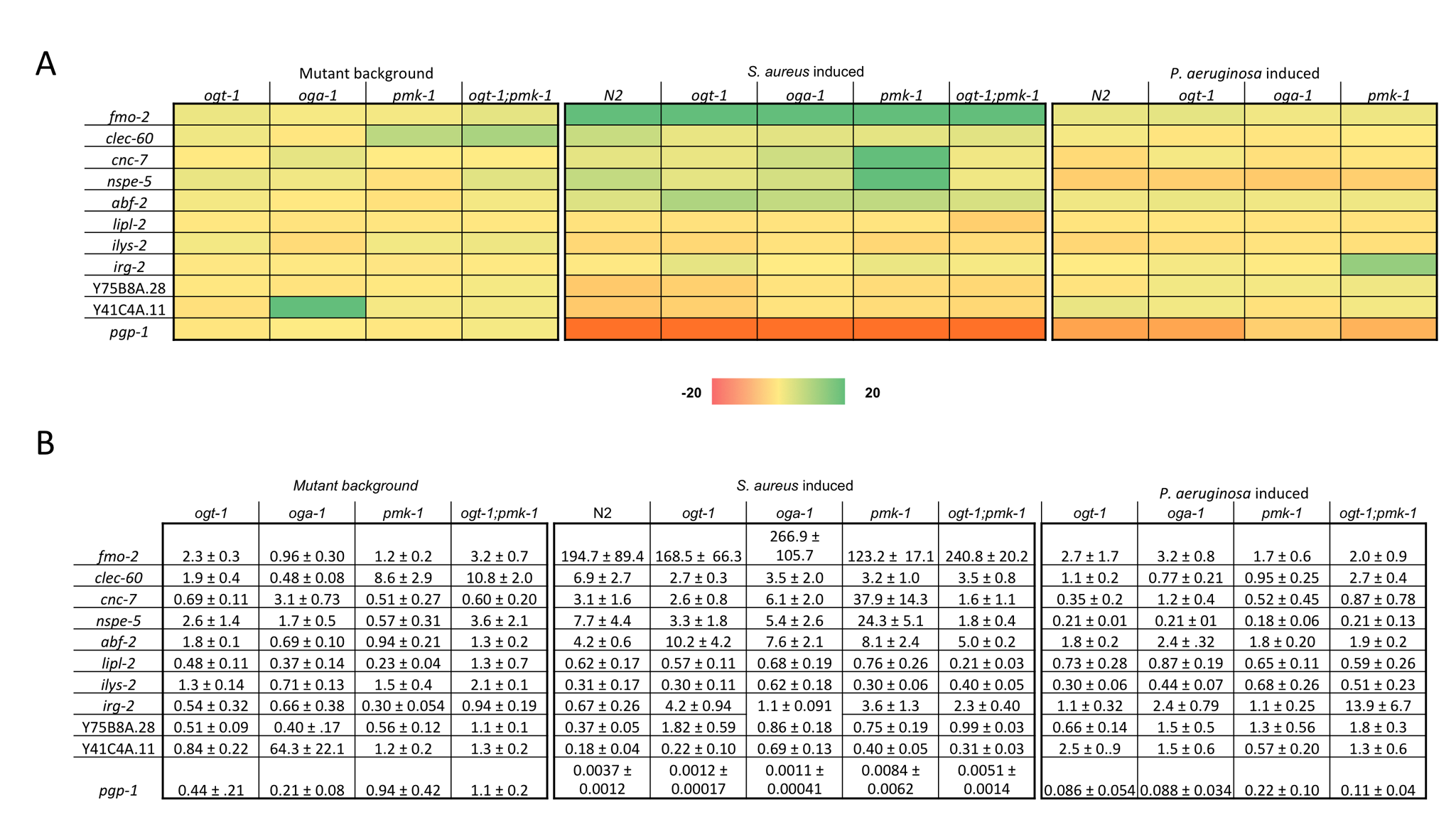

Supplement: Figure S5 — Genes are differentially induced in N2 and mutant worms upon S. aureus exposure. RT-qPCR analysis shows the induction or repression of selected genes: fmo-2, clec-60, cnc-7, nspe-5, abf-2, lipl-2, ilys-2, irg-2, Y75B8A.23, Y41C4A.11, and pgp-1. Data are the relative levels of expression of genes differentially regulated in mutant backgrounds compared to N2 on OP50 and those deregulated within an individual mutant upon pathogen exposure. Data in the heat map (A) represent the means of three biological replicates and are normalized to the geometric mean of two housekeeping genes (snb-1 and gpd-3) (B). N2, ogt-1(ok1474), oga-1(ok1207), pmk-1(km25), and ogt-1(ok1474); pmk-1(km25) strains were used. (TIF) [file pone.0113231.s005.tif]

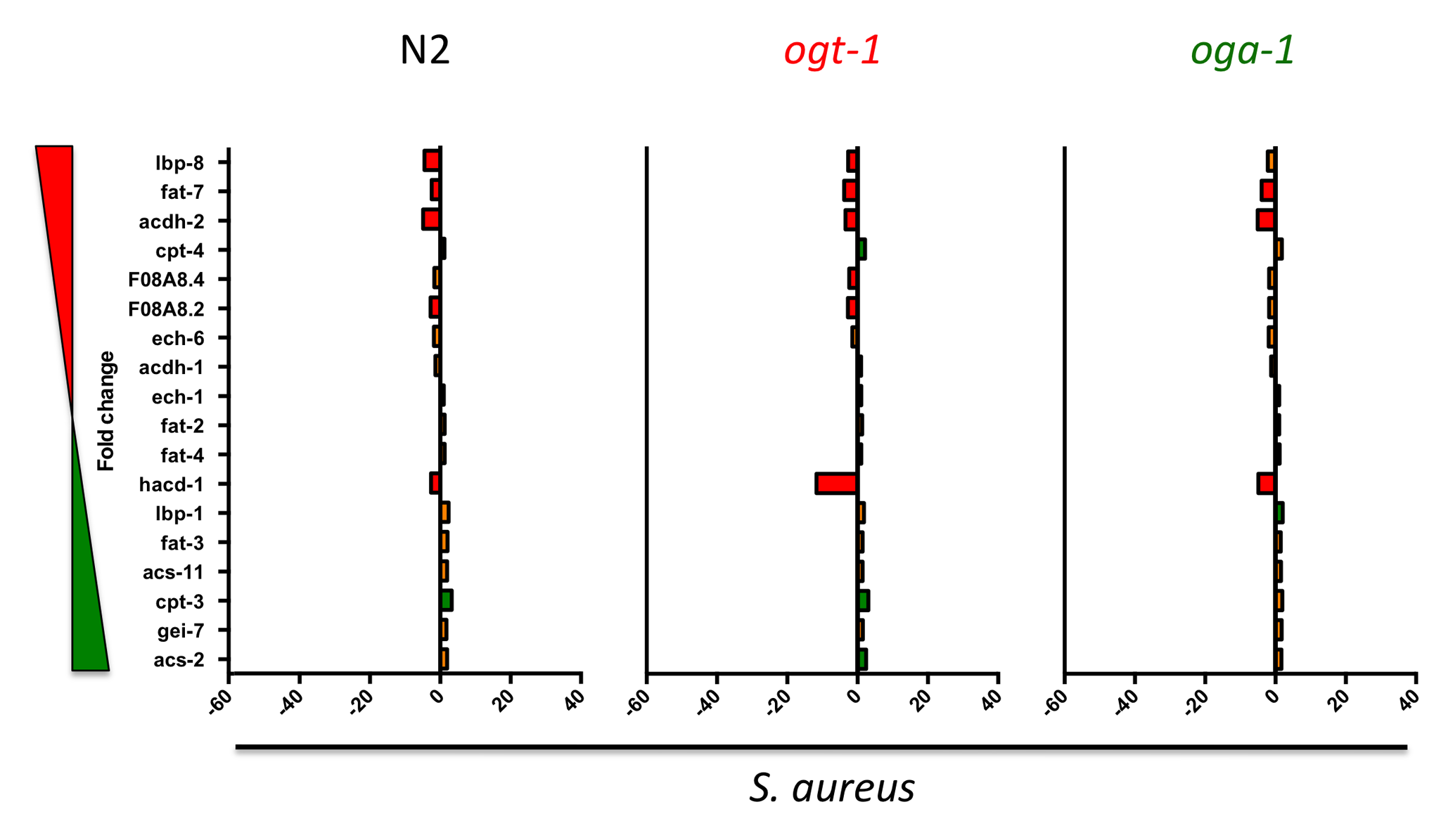

Supplement: Figure S6 — S. aureus exposed animals do not undergo starvation response. 18 fasting response genes were identified by qRT-PCR by Van Gilst and colleagues [31]. Maximum fold repression in starved L4 ranged from −1.6 (ech-1) to −55.0 (lbp-8) (red triangle) while maximum fold induction in starved L4 ranged from 2.8 (fat-2) to 39.8 (acs-2) (green triangle). The transcriptional responses in N2, ogt-1(ok1474), and oga-1(ok1207) animals fed S. aureus were compared to the same genotypes fed OP50 by microarray and changes were generally negligible. Bars highlighted in green indicate increases in gene expression while bars highlighted in red indicate decreases in gene expression over 2-fold. Orange bars represent gene expression changes <2-fold. (TIF) [file pone.0113231.s006.tif]

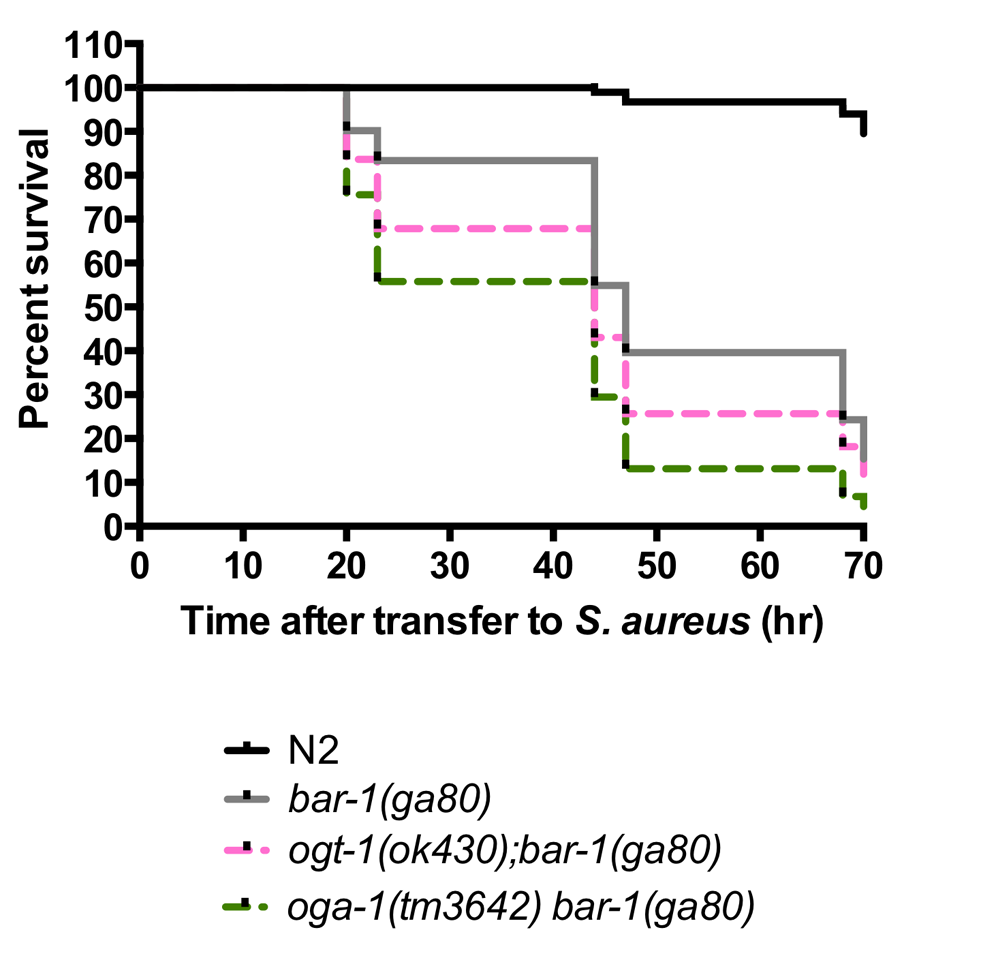

Supplement: Figure S7 — Survival of bar-1 single and double mutants after 48 hr cdc25.1 RNAi exposure. bar-1(ga80) single mutants, ogt-1(ok430); bar-1(ga80), and oga-1(tm3642) bar-1(ga80) double mutants have similar survivals. N2 appears to have a lengthened survival likely due to persistent cdc25.1 RNAi presence. Killing assays were performed with S. aureus under conditions described in Materials and Methods and are represented here by comprehensive plots. n≧154. Statistics for all experiments are available in Table S1. (TIF) [file pone.0113231.s007.tif]
