# Supplementary material for: Conserved Nutrient Sensor O-GlcNAc Transferase Is Integral to C. elegans Pathogen-Specific Immunity
Source: PLoS One. 2014 Dec 4;9(12):e113231. doi: 10.1371/journal.pone.0113231 (PMC4256294; doi:10.1371/journal.pone.0113231)
Supplement: Table S1 — Lifespan statistics. (PDF) [file pone.0113231.s008.pdf]

Table S1. Lifespan statistics for animals fed OP50, *S. aureus*, and *P. aerugionsa*

Experiment 1: OP50 fed				
	N	n, censor	Median Survival	P value logrank (vs. N2)
N2	89	1	386	
ogt-1(ok430)	84	7	482	<0.0001
ogt-1(ok1474)	67	23	410	0.0008
oga- (ok1207)	65	27	482	<0.0001
oga-1(tm3642)	78	12	386	0.0009
pmk-1(km25)	73	17	386	0.5155
ogt1 (ok1474); oga-1 (ok1207)	79	11	386	0.512
ogt-1(ok1474); pmk-1(km25)	59	31	356	0.1396
pmk-1(km25); oga-1(ok1207)	58	32	386	0.0978
bar-1(ga80)	69	21	166	<0.0001
ogt-1(ok1474);bar-1(ga80)	32	20	96	<0.0001
oga-1(ok1207) bar-1(ga80)	57	34	144	<0.0001

Experiment 2: OP50 fed				
	N	n, censor	Median Survival	P value logrank (vs. N2)
N2	57	3	378	
ogt-1(ok430)	66	24	455	0.0009
oga-1(tm3642)	67	23	378	0.7857
pmk-1(km25)	76	14	378	0.1668
ogt-1(ok430); pmk-1(km25)	74	16	455	0.0066
pmk-1(km25); oga-1(tm3642)	69	21	408	0.6035

Experiment 3: OP50 fed				
	N	n, censor	Median Survival	P value logrank (vs. N2)
N2	118	7	357	
ogt-1(ok430)	78	14	451	<0.0001
oga-1(tm3642)	114	6	379	0.4712
pmk-1(km25)	100	20	357	0.994
ogt-1(ok430); pmk-1(km25)	69	22	451	<0.0001
pmk-1(km25); oga-1(tm3642)	107	14	451	0.0068

Experiment 4: OP50 fed				
	N	n, censor	Median Survival	P value logrank (vs. N2)
N2	86	4	360	
ogt-1(ok1474)	76	14	410	0.0014
oga- (ok1207)	69	21	410	0.001
pmk-1(km25)	80	20	360	0.0143
ogt-1(ok1474); oga-1 (ok1207)	98	8	385	0.2797
ogt-1(ok1474); pmk-1(km25)	93	22	385	0.6879
pmk-1(km25); oga-1(ok1207)	90	20	385	0.0262

Experiment 5: OP50 fed				
	N	n, censor	Median Survival	P value logrank (vs. N2)
N2	144	6	384	
bar-1(ga80)	122	18	140	<0.0001
ogt-1(ok430);bar-1(ga80)	128	18	68	<0.0001
ogt-1(ok1474);bar-1(ga80)	135	19	96	<0.0001
oga-1(ok1207) bar-1(ga80)	144	7	120	<0.0001
oga-1(tm3642) bar-1(ga80)	134	4	68	<0.0001

Experiment 6: OP50 fed				
	N	n, censor	Median Survival	P value logrank (vs. N2)
N2	50	9	383	
bar-1(ga80)	60	21	146	<0.0001
ogt-1(ok430);bar-1(ga80)	79	17	74	<0.0001
ogt-1(ok1474);bar-1(ga80)	87	5	74	<0.0001
oga-1(ok1207) bar-1(ga80)	78	12	122	<0.0001
oga-1(tm3642) bar-1(ga80)	78	9	50	<0.0001

Experiment 1: P. aeruginosa exposure				
	N	n, censor	Median Survival	P value logrank (vs. N2)
N2	87	2	144	
ogt-1(ok430)	85	4	150	<0.0001
ogt-1(ok1474)	76	19	144	0.0061
oga- (ok1207)	95	1	144	0.0318
oga-1(tm3642)	85	2	144	0.3393
pmk-1(km25)	75	9	96	<0.0001
ogt1 (ok1474); oga-1(ok1207)	97	0	144	0.2535
ogt-1(ok430); pmk-1(km25)	94	4	96	<0.0001
ogt-1(ok1474); pmk-1(km25)	89	4	96	<0.0001
pmk-1(km25); oga-1(ok1207)	96	0	96	<0.0001
pmk-1(km25); oga-1(tm3642)	92	3	96	<0.0001

Experiment 2: P. aeruginosa exposure				
	N	n, censor	Median Survival	P value logrank (vs. N2)
N2	90	1	93	
ogt-1(ok430)	104	6	117	<0.0001
ogt-1(ok1474)	96	4	117	<0.0001
oga- (ok1207)	105	2	117	<0.0001
oga-1(tm3642)	67	10	117	0.0101
pmk-1(km25)	108	4	68	<0.0001
ogt1 (ok1474); oga-1(ok1207)	79	14	117	<0.0001
ogt-1(ok430); pmk-1(km25)	76	12	68	<0.0001
ogt-1(ok1474); pmk-1(km25)	93	2	68	<0.0001
pmk-1(km25); oga-1(ok1207)	86	3	68	<0.0001
pmk-1(km25); oga-1(tm3642)	86	6	45	<0.0001

Experiment 3: P. aeruginosa exposure				
	N	n, censor	Median Survival	P value logrank (vs. N2)
N2	97	0	123	
ogt-1(ok430)	100	6	123	0.0333
ogt-1(ok1474)	102	5	123	0.0592
oga- (ok1207)	104	6	123	0.0541
oga-1(tm3642)	75	21	123	0.0434
pmk-1(km25)	95	0	50	<0.0001
ogt1 (ok1474); oga-1(ok1207)	104	6	123	0.0421
ogt-1(ok430); pmk-1(km25)	107	2	73	<0.0001
ogt-1(ok1474); pmk-1(km25)	103	0	50	<0.0001
pmk-1(km25); oga-1(ok1207)	97	1	73	<0.0001
pmk-1(km25); oga-1(tm3642)	105	2	50	<0.0001

Experiment 1: S. aureus exposure				
	N	n, censor	Median Survival	P value logrank (vs. N2)
N2	107	13	50	
ogt-1(ok430)	104	11	42	<0.0001
oga-1(tm3642)	113	7	50	0.5224
pmk-1(km25)	108	7	42	<0.0001
ogt-1(ok430); pmk-1(km25)	95	8	28	<0.0001
pmk-1(km25); oga-1(tm3642)	114	8	42	<0.0001

Experiment 2: S. aureus exposure				
	N	n, censor	Median Survival	P value logrank (vs. N2)
N2	15	0	74	
ogt-1(ok1474)	19	1	44	0.0078
oga- (ok1207)	20	0	74	0.1839

Experiment 3: S. aureus exposure				
	N	n, censor	Median Survival	P value logrank (vs. N2)
N2	74	16	65	
ogt-1(ok430)	89	0	42	<0.0001
ogt-1(ok1474)	90	0	24	<0.0001
oga- (ok1207)	84	0	49	0.0052

oga-1(tm3642)	75	15	49	0.0241
pmk-1(km25)	73	2	20	<0.0001
ogt1 (ok1474); oga-1(ok1207)	87	0	42	<0.0001
ogt-1(ok430); pmk-1(km25)	67	13	20	<0.0001
ogt-1(ok1474); pmk-1(km25)	73	7	20	<0.0001
pmk-1(km25); oga-1(ok1207)	85	5	24	<0.0001
pmk-1(km25); oga-1(tm3642)	73	5	42	<0.0001

Experiment 4: S. aureus exposure				
	N	n, censor	Median Survival	P value logrank (vs. N2)
N2	82	9	50	
ogt-1(ok430)	86	4	31	<0.0001
ogt-1(ok1474)	92	0	31	<0.0001
oga- (ok1207)	91	0	50	0.3773
oga-1(tm3642)	86	6	45.5	0.8152
pmk-1(km25)	89	5	26	<0.0001
bar-1(ga80)	81	8	26	<0.0001

Experiment 5: S. aureus exposure				
	N	n, censor	Median Survival	P value logrank (vs. N2)
N2	42	51	50	
ogt-1(ok430)	73	22	50	<0.0001
ogt-1(ok1474)	77	23	50	0.0075
oga- (ok1207)	54	41	50	0.451
oga-1(tm3642)	48	49	50	0.8194
pmk-1(km25)	51	49	41	0.0231
bar-1(ga80)	104	21	41	<0.0001

Experiment 6: S. aureus exposure				
	N	n, censor	Median Survival	P value logrank (vs. N2)
N2	113	10	50	
ogt-1(ok430)	103	7	42	<0.0001
ogt-1(ok1474)	109	3	42	<0.0001
oga- (ok1207)	101	9	50	0.2529
oga-1(tm3642)	105	5	50	0.6217
pmk-1(km25)	106	6	42	<0.0001
ogt1 (ok1474); oga-1(ok1207)	110	3	42	<0.0001
bar-1(ga80)	96	14	30	<0.0001
ogt-1(ok1474);bar-1(ga80)	24	43	42	<0.0001
oga-1(ok1207) bar-1(ga80)	84	11	42	<0.0001

Experiment 7: S. aureus exposure				
	N	n, censor	Median Survival	P value logrank (vs. N2)
N2	103	13	50	
ogt-1(ok430)	103	11	46	<0.0001
ogt-1(ok1474)	94	7	40	<0.0001
oga- (ok1207)	113	3	50	0.3144
oga-1(tm3642)	110	10	50	0.7666
pmk-1(km25)	99	21	40	<0.0001
ogt1 (ok1474); oga-1(ok1207)	100	5	46	<0.0001
ogt-1(ok1474); pmk-1(km25)	89	16	26	<0.0001
pmk-1(km25); oga-1(ok1207)	91	14	30	<0.0001
bar-1(ga80)	82	23	40	<0.0001
ogt-1(ok1474);bar-1(ga80)	33	21	40	<0.0001
oga-1(ok1207) bar-1(ga80)	56	39	40	<0.0001

Experiment 8: S. aureus exposure *48 hr RNAi feeding				
	N	n, censor	Median Survival	P value logrank (vs. N2)
N2	83	21	356	
bar-1(ga80)	124	1	44	< 0.0001
ogt-1(ok430);bar-1(ga80)	122	8	44	< 0.0001
ogt-1(ok1474);bar-1(ga80)	151	9	44	< 0.0001
oga-1(ok1207) bar-1(ga80)	118	0	44	< 0.0001
oga-1(tm3642) bar-1(ga80)	134	1	44	< 0.0001

Experiment 9: S. aureus exposure *48 hr RNAi feeding				
	N	n, censor	Median Survival	P value logrank (vs. N2)
N2	71	9	289	
bar-1(ga80)	66	2	47	< 0.0001
ogt-1(ok430);bar-1(ga80)	101	32	47	< 0.0001
ogt-1(ok1474);bar-1(ga80)	78	20	47	< 0.0001
oga-1(ok1207) bar-1(ga80)	71	2	47	< 0.0001
oga-1(tm3642) bar-1(ga80)	90	4	47	< 0.0001
