# Supplementary material for: Conserved Nutrient Sensor O-GlcNAc Transferase Is Integral to C. elegans Pathogen-Specific Immunity
Source: PLoS One. 2014 Dec 4;9(12):e113231. doi: 10.1371/journal.pone.0113231 (PMC4256294; doi:10.1371/journal.pone.0113231)
Supplement: Table S2 — Pumping rate statistics. (PDF) [file pone.0113231.s009.pdf]

Table S2. Pumping rate statistics

Alpha	0.01											
	Mean Diff.	Significant?	Summary	Individual P Value	Mean 1	Mean 2	Mean Diff.	SE of diff.	n1	n2	t	DF
N2 OP50 vs. ok430 OP50	7.125	No	ns	0.2291	78.94	71.81	7.125	5.915	16	16	1.204	422
N2 OP50 vs. ok1474 OP50	3.392	No	ns	0.605	78.94	75.55	3.392	6.553	16	11	0.5176	422
N2 OP50 vs. ok1207 OP50	4.574	No	ns	0.4856	78.94	74.36	4.574	6.553	16	11	0.698	422
N2 OP50 vs. tm3642 OP50	2.375	No	ns	0.6883	78.94	76.56	2.375	5.915	16	16	0.4015	422
N2 OP50 vs. km25 OP50	2	No	ns	0.7355	78.94	76.94	2	5.915	16	16	0.3381	422
N2 OP50 vs. ok1474;ok1207 OP50	4.483	No	ns	0.4943	78.94	74.45	4.483	6.553	16	11	0.6841	422
N2 OP50 vs. ok430;km25 OP50	12.39	No	ns	0.0593	78.94	66.55	12.39	6.553	16	11	1.891	422
N2 OP50 vs. ok1474;km25 OP50	8.301	No	ns	0.206	78.94	70.64	8.301	6.553	16	11	1.267	422
N2 OP50 vs. ok1207;km25 OP50	4.483	No	ns	0.4943	78.94	74.45	4.483	6.553	16	11	0.6841	422
N2 OP50 vs. tm3642;km25 OP50	6.665	No	ns	0.3097	78.94	72.27	6.665	6.553	16	11	1.017	422
N2 OP50 vs. N2 SA	25.38	Yes	****	< 0.0001	78.94	53.56	25.38	5.749	16	18	4.415	422
N2 OP50 vs. ok430 SA	48.14	Yes	****	< 0.0001	78.94	30.8	48.14	5.357	16	25	8.987	422
N2 OP50 vs. ok1474 SA	41.07	Yes	****	< 0.0001	78.94	37.87	41.07	6.013	16	15	6.83	422
N2 OP50 vs. ok1207 SA	24.85	Yes	***	0.0001	78.94	54.08	24.85	6.389	16	12	3.89	422
N2 OP50 vs. tm3642 SA	24.05	Yes	****	< 0.0001	78.94	54.89	24.05	5.749	16	18	4.183	422
N2 OP50 vs. km25 SA	36.53	Yes	****	< 0.0001	78.94	42.41	36.53	5.497	16	22	6.645	422
N2 OP50 vs. ok1474;ok1207 SA	48.56	Yes	****	< 0.0001	78.94	30.38	48.56	5.915	16	16	8.21	422
N2 OP50 vs. ok430;km25 SA	71.39	Yes	****	< 0.0001	78.94	7.545	71.39	6.553	16	11	10.89	422
N2 OP50 vs. ok1474;km25 SA	69.14	Yes	****	< 0.0001	78.94	9.8	69.14	6.745	16	10	10.25	422
N2 OP50 vs. ok1207;km25 SA	40.17	Yes	****	< 0.0001	78.94	38.77	40.17	6.247	16	13	6.43	422
N2 OP50 vs. tm3642;km25 SA	43.44	Yes	****	< 0.0001	78.94	35.5	43.44	5.915	16	16	7.343	422
N2 OP50 vs. N2 PA	12.64	No	ns	0.0617	78.94	66.3	12.64	6.745	16	10	1.874	422
N2 OP50 vs. ok430 PA	13.74	No	*	0.0423	78.94	65.2	13.74	6.745	16	10	2.037	422
N2 OP50 vs. ok1474 PA	15.84	No	*	0.0193	78.94	63.1	15.84	6.745	16	10	2.348	422
N2 OP50 vs. ok1207 PA	25.34	Yes	***	0.0002	78.94	53.6	25.34	6.745	16	10	3.757	422
N2 OP50 vs. tm3642 PA	29.64	Yes	****	< 0.0001	78.94	49.3	29.64	6.745	16	10	4.394	422
N2 OP50 vs. km25 PA	42.54	Yes	****	< 0.0001	78.94	36.4	42.54	6.745	16	10	6.307	422
N2 OP50 vs. ok1474;ok1207 PA	20.34	Yes	**	0.0027	78.94	58.6	20.34	6.745	16	10	3.015	422
N2 OP50 vs. ok430;km25 PA	59.34	Yes	****	< 0.0001	78.94	19.6	59.34	6.745	16	10	8.798	422
N2 OP50 vs. ok1474;km25 PA	39.54	Yes	****	< 0.0001	78.94	39.4	39.54	6.745	16	10	5.862	422
N2 OP50 vs. ok1207;km25 PA	42.64	Yes	****	< 0.0001	78.94	36.3	42.64	6.745	16	10	6.322	422

N2 OP50 vs. tm3642;km25 PA	53.04	Yes	****	< 0.0001	78.94	25.9	53.04	6.745	16	10	7.864	422
ok430 OP50 vs. ok430;km25 OP50	5.267	No	ns	0.422	71.81	66.55	5.267	6.553	16	11	0.8037	422
ok430 OP50 vs. ok430 SA	41.01	Yes	****	< 0.0001	71.81	30.8	41.01	5.357	16	25	7.656	422
ok430 OP50 vs. ok430 PA	6.613	No	ns	0.3274	71.81	65.2	6.613	6.745	16	10	0.9804	422
ok1474 OP50 vs. ok1474;ok1207 OP50	1.091	No	ns	0.8785	75.55	74.45	1.091	7.134	11	11	0.1529	422
ok1474 OP50 vs. ok1474;km25 OP50	4.909	No	ns	0.4918	75.55	70.64	4.909	7.134	11	11	0.6881	422
ok1474 OP50 vs. ok1474 SA	37.68	Yes	****	< 0.0001	75.55	37.87	37.68	6.642	11	15	5.673	422
ok1474 OP50 vs. ok1474 PA	12.45	No	ns	0.0894	75.55	63.1	12.45	7.31	11	10	1.702	422
ok1207 OP50 vs. ok1474;ok1207 OP50	-0.09091	No	ns	0.9898	74.36	74.45	-0.09091	7.134	11	11	0.01274	422
ok1207 OP50 vs. ok1207;km25 OP50	-0.09091	No	ns	0.9898	74.36	74.45	-0.09091	7.134	11	11	0.01274	422
ok1207 OP50 vs. ok1207 SA	20.28	Yes	**	0.0039	74.36	54.08	20.28	6.984	11	12	2.904	422
ok1207 OP50 vs. ok1207 PA	20.76	Yes	**	0.0047	74.36	53.6	20.76	7.31	11	10	2.84	422
tm3642 OP50 vs. tm3642;km25 OP50	4.29	No	ns	0.5131	76.56	72.27	4.29	6.553	16	11	0.6546	422
tm3642 OP50 vs. tm3642 SA	21.67	Yes	***	0.0002	76.56	54.89	21.67	5.749	16	18	3.77	422
tm3642 OP50 vs. tm3642 PA	27.26	Yes	****	< 0.0001	76.56	49.3	27.26	6.745	16	10	4.042	422
km25 OP50 vs. ok430;km25 OP50	10.39	No	ns	0.1135	76.94	66.55	10.39	6.553	16	11	1.586	422
km25 OP50 vs. ok1474;km25 OP50	6.301	No	ns	0.3368	76.94	70.64	6.301	6.553	16	11	0.9615	422
km25 OP50 vs. ok1207;km25 OP50	2.483	No	ns	0.705	76.94	74.45	2.483	6.553	16	11	0.3789	422
km25 OP50 vs. tm3642;km25 OP50	4.665	No	ns	0.477	76.94	72.27	4.665	6.553	16	11	0.7118	422
km25 OP50 vs. km25 SA	34.53	Yes	****	< 0.0001	76.94	42.41	34.53	5.497	16	22	6.281	422
km25 OP50 vs. km25 PA	40.54	Yes	****	< 0.0001	76.94	36.4	40.54	6.745	16	10	6.01	422
ok1474;ok1207 OP50 vs. ok1474;ok1207 SA	44.08	Yes	****	< 0.0001	74.45	30.38	44.08	6.553	11	16	6.726	422
ok1474;ok1207 OP50 vs. ok1474;ok1207 PA	15.85	No	*	0.0307	74.45	58.6	15.85	7.31	11	10	2.169	422
ok430;km25 OP50 vs. ok430;km25 SA	59	Yes	****	< 0.0001	66.55	7.545	59	7.134	11	11	8.27	422
ok430;km25 OP50 vs. ok430;km25 PA	46.95	Yes	****	< 0.0001	66.55	19.6	46.95	7.31	11	10	6.422	422
ok1474;km25 OP50 vs. ok1474;km25 SA	60.84	Yes	****	< 0.0001	70.64	9.8	60.84	7.31	11	10	8.322	422
ok1474;km25 OP50 vs. ok1474;km25 PA	31.24	Yes	****	< 0.0001	70.64	39.4	31.24	7.31	11	10	4.273	422
ok1207;km25 OP50 vs. ok1207;km25 SA	35.69	Yes	****	< 0.0001	74.45	38.77	35.69	6.854	11	13	5.206	422
ok1207;km25 OP50 vs. ok1207;km25 PA	38.15	Yes	****	< 0.0001	74.45	36.3	38.15	7.31	11	10	5.219	422
tm3642;km25 OP50 vs. tm3642;km25 SA	36.77	Yes	****	< 0.0001	72.27	35.5	36.77	6.553	11	16	5.611	422
tm3642;km25 OP50 vs. tm3642;km25 PA	46.37	Yes	****	< 0.0001	72.27	25.9	46.37	7.31	11	10	6.343	422
N2 SA vs. ok430 SA	22.76	Yes	****	< 0.0001	53.56	30.8	22.76	5.172	18	25	4.4	422
N2 SA vs. ok1474 SA	15.69	Yes	**	0.0076	53.56	37.87	15.69	5.849	18	15	2.682	422
N2 SA vs. ok1207 SA	-0.5278	No	ns	0.9326	53.56	54.08	-0.5278	6.235	18	12	0.08464	422
N2 SA vs. tm3642 SA	-1.333	No	ns	0.8112	53.56	54.89	-1.333	5.577	18	18	0.2391	422
N2 SA vs. km25 SA	11.15	No	*	0.0367	53.56	42.41	11.15	5.318	18	22	2.096	422
N2 SA vs. ok1474;ok1207 SA	23.18	Yes	****	< 0.0001	53.56	30.38	23.18	5.749	18	16	4.032	422

N2 SA vs. ok430;km25 SA	46.01	Yes	****	< 0.0001	53.56	7.545	46.01	6.403	18	11	7.186	422
N2 SA vs. ok1474;km25 SA	43.76	Yes	****	< 0.0001	53.56	9.8	43.76	6.599	18	10	6.631	422
N2 SA vs. ok1207;km25 SA	14.79	No	*	0.0156	53.56	38.77	14.79	6.09	18	13	2.428	422
N2 SA vs. tm3642;km25 SA	18.06	Yes	**	0.0018	53.56	35.5	18.06	5.749	18	16	3.141	422
ok430 SA vs. ok430;km25 SA	23.25	Yes	***	0.0001	30.8	7.545	23.25	6.054	25	11	3.841	422
ok1474 SA vs. ok1474;ok1207 SA	7.492	No	ns	0.2135	37.87	30.38	7.492	6.013	15	16	1.246	422
ok1474 SA vs. ok1474;km25 SA	28.07	Yes	****	< 0.0001	37.87	9.8	28.07	6.83	15	10	4.109	422
ok1207 SA vs. ok1474;ok1207 SA	23.71	Yes	***	0.0002	54.08	30.38	23.71	6.389	12	16	3.711	422
ok1207 SA vs. ok1207;km25 SA	15.31	No	*	0.0227	54.08	38.77	15.31	6.698	12	13	2.286	422
tm3642 SA vs. tm3642;km25 SA	19.39	Yes	***	0.0008	54.89	35.5	19.39	5.749	18	16	3.373	422
km25 SA vs. ok430;km25 SA	34.86	Yes	****	< 0.0001	42.41	7.545	34.86	6.178	22	11	5.643	422
km25 SA vs. ok1474;km25 SA	32.61	Yes	****	< 0.0001	42.41	9.8	32.61	6.381	22	10	5.11	422
km25 SA vs. ok1207;km25 SA	3.64	No	ns	0.5344	42.41	38.77	3.64	5.853	22	13	0.6219	422
km25 SA vs. tm3642;km25 SA	6.909	No	ns	0.2095	42.41	35.5	6.909	5.497	22	16	1.257	422
N2 PA vs. ok430 PA	1.1	No	ns	0.8832	66.3	65.2	1.1	7.482	10	10	0.147	422
N2 PA vs. ok1474 PA	3.2	No	ns	0.6691	66.3	63.1	3.2	7.482	10	10	0.4277	422
N2 PA vs. ok1207 PA	12.7	No	ns	0.0904	66.3	53.6	12.7	7.482	10	10	1.697	422
N2 PA vs. tm3642 PA	17	No	*	0.0236	66.3	49.3	17	7.482	10	10	2.272	422
N2 PA vs. km25 PA	29.9	Yes	****	< 0.0001	66.3	36.4	29.9	7.482	10	10	3.996	422
N2 PA vs. ok1474;ok1207 PA	7.7	No	ns	0.304	66.3	58.6	7.7	7.482	10	10	1.029	422
N2 PA vs. ok430;km25 PA	46.7	Yes	****	< 0.0001	66.3	19.6	46.7	7.482	10	10	6.241	422
N2 PA vs. ok1474;km25 PA	26.9	Yes	***	0.0004	66.3	39.4	26.9	7.482	10	10	3.595	422
N2 PA vs. ok1207;km25 PA	30	Yes	****	< 0.0001	66.3	36.3	30	7.482	10	10	4.009	422
N2 PA vs. tm3642;km25 PA	40.4	Yes	****	< 0.0001	66.3	25.9	40.4	7.482	10	10	5.399	422
ok430 PA vs. ok430;km25 PA	45.6	Yes	****	< 0.0001	65.2	19.6	45.6	7.482	10	10	6.094	422
ok1474 PA vs. ok1474;ok1207 PA	4.5	No	ns	0.5479	63.1	58.6	4.5	7.482	10	10	0.6014	422
ok1474 PA vs. ok1474;km25 PA	23.7	Yes	**	0.0016	63.1	39.4	23.7	7.482	10	10	3.167	422
ok1207 PA vs. ok1474;ok1207 PA	-5	No	ns	0.5044	53.6	58.6	-5	7.482	10	10	0.6682	422
ok1207 PA vs. ok1207;km25 PA	17.3	No	*	0.0213	53.6	36.3	17.3	7.482	10	10	2.312	422
tm3642 PA vs. tm3642;km25 PA	23.4	Yes	**	0.0019	49.3	25.9	23.4	7.482	10	10	3.127	422
km25 PA vs. ok430;km25 PA	16.8	No	*	0.0253	36.4	19.6	16.8	7.482	10	10	2.245	422
km25 PA vs. ok1474;km25 PA	-3	No	ns	0.6887	36.4	39.4	-3	7.482	10	10	0.4009	422
km25 PA vs. ok1207;km25 PA	0.1	No	ns	0.9893	36.4	36.3	0.1	7.482	10	10	0.01336	422
km25 PA vs. tm3642;km25 PA	10.5	No	ns	0.1613	36.4	25.9	10.5	7.482	10	10	1.403	422
