# Supplementary material for: Conserved Nutrient Sensor O-GlcNAc Transferase Is Integral to C. elegans Pathogen-Specific Immunity
Source: PLoS One. 2014 Dec 4;9(12):e113231. doi: 10.1371/journal.pone.0113231 (PMC4256294; doi:10.1371/journal.pone.0113231)
Supplement: Table S3 — Broodsize statistics. (PDF) [file pone.0113231.s010.pdf]

Table S3. Broodsize statistics

Alpha	0.001							
	Mean Diff.	Significant?	Summary	Individual P Value				
N2 vs. ok1474	20.67	No	**	0.0069				
N2 vs. ok430	30.51	Yes	***	0.0005				
N2 vs. ok1207	0.1143	No	ns	0.988				
N2 vs. tm3642	-6.849	No	ns	0.4347				
N2 vs. km25	2.901	No	ns	0.6639				
Test details	Mean 1	Mean 2	Mean Diff.	SE of diff.	n1	n2	t	DF
N2 vs. ok1474	209	188.4	20.67	7.621	63	45	2.712	558
N2 vs. ok430	209	178.5	30.51	8.661	63	30	3.523	558
N2 vs. ok1207	209	208.9	0.1143	7.621	63	45	0.015	558
N2 vs. tm3642	209	215.9	-6.849	8.762	63	29	0.7817	558
N2 vs. km25	209	206.1	2.901	6.673	63	75	0.4347	558

Alpha	0.001							
	Mean Diff.	Significant?	Summary	Individual P Value				
N2 vs. ga80	83.37	Yes	****	< 0.0001				
Test details	Mean 1	Mean 2	Mean Diff.	SE of diff.	n1	n2	t	DF
N2 vs. ga80	195	111.7	83.37	10.64	33	38	7.837	217
