# Supplementary material for: Conserved Nutrient Sensor O-GlcNAc Transferase Is Integral to C. elegans Pathogen-Specific Immunity
Source: PLoS One. 2014 Dec 4;9(12):e113231. doi: 10.1371/journal.pone.0113231 (PMC4256294; doi:10.1371/journal.pone.0113231)
Supplement: Table S4 — Genes enriched in ogt-1, oga-1, and pmk-1 mutants by microarray. (PDF) [file pone.0113231.s011.pdf]

Table S4. Categories of genes identified as enriched by DAVID analysis of microarray samples of L4 animals comparing *ogt-1(ok1474), oga-1(ok1207), or pmk-1(km25)* with N2 on OP50. Functional annotation clustering was performed using DAVID default settings and highlights biological pathways enriched more than 2 fold in each set with $p < 0.05$.

***ogt-1(ok1474)* enriched**		***oga-1(ok1207)* enriched**		***pmk-1(km25)* enriched**	
Category	Enrichment score	Category	Enrichment score	Category	Enrichment score
cysteine-rich repeat	6.35	collagen	11.43	collagen	15.1
pan-1 domain/apple-like	4.67	molting cycle and multicellular organism growth	9.45	sterol-sensing	9.48
carbohydrate binding	4.03	sterol-sensing	9.45	molting cycleand multicellular organism growth	7.56
collagen	3.92	pan-1 domain	6.71	signal peptide/glycoprotein	7.2
signal peptide and glycoprotein	3.58	cuticle development	4.95	cuticle development	7.19
cytochrome p450	2.96	protein splicing	4.44	cysteine-rich repeat	7.16
proteolysis	2.67	protease	3.57	pan-1 domain	5.03
protein maturation and processing	2.86	carbohydrate binding	3.47	carbohydrate binding	4.84
glycosidase/chitin processing	2.5	cysteine-rich repeat	3.06	protein splicing	4.1
molting and development	2.33	protein of unknown function	2.57	protease	3.38
lipid binding	2.26	cytochrome p450	2.06	c-type lectin	3.21
protein of unknown function	2.14			polysaccharide metabolic process	3.13
				enzyme inhibitor activity	2.86
